# Estimating effective population size changes from preferentially sampled genetic sequences

**DOI:** 10.1371/journal.pcbi.1007774

**Published:** 2020-10-12

**Authors:** Michael D. Karcher, Luiz Max Carvalho, Marc A. Suchard, Gytis Dudas, Vladimir N. Minin

**Affiliations:** 1 Department of Statistics, University of Washington, Seattle, Washington, U.S.A.; 2 National School of Public Health, Oswaldo Cruz Foundation, Brazil; 3 Department of Human Genetics, David Geffen School of Medicine at UCLA, University of California, Los Angeles, California, U.S.A.; 4 Department of Biomathematics, David Geffen School of Medicine at UCLA, University of California, Los Angeles, California, U.S.A.; 5 Department of Biostatistics, UCLA Fielding School of Public Health, University of California, Los Angeles, California, U.S.A.; 6 Vaccine and Infectious Disease Division, Fred Hutchinson Cancer Research Center; 7 Gothenburg Global Biodiversity Centre (GGBC), Gothenburg, Sweden; 8 Department of Statistics, University of California, Irvine, California, U.S.A.; Temple University, UNITED STATES

## Abstract

Coalescent theory combined with statistical modeling allows us to estimate effective population size fluctuations from molecular sequences of individuals sampled from a population of interest. When sequences are sampled serially through time and the distribution of the sampling times depends on the effective population size, explicit statistical modeling of sampling times improves population size estimation. Previous work assumed that the genealogy relating sampled sequences is known and modeled sampling times as an inhomogeneous Poisson process with log-intensity equal to a linear function of the log-transformed effective population size. We improve this approach in two ways. First, we extend the method to allow for joint Bayesian estimation of the genealogy, effective population size trajectory, and other model parameters. Next, we improve the sampling time model by incorporating additional sources of information in the form of time-varying covariates. We validate our new modeling framework using a simulation study and apply our new methodology to analyses of population dynamics of seasonal influenza and to the recent Ebola virus outbreak in West Africa.

## Author summary

Estimating changes in the number of individuals in a given population is a challenging problem in some settings. For example, estimating population size trajectories of the number of people infected by a pathogen (e.g., Influenza virus) is a difficult problem, because many infections in a large population remain unobserved/hidden. One indirect way of assessing population size changes is to take a sample of individuals from the population of interest and analyze genetic sequences from these individuals (e.g., Influenza virus genomes). Intuitively, genetic data is informative about population size changes, because genetic diversity increases/decreases together with the population size. However, if we sample more individuals when the population size increases and less when it decreases, this strategy produces biased results. To avoid this bias, we propose a method that explicitly and flexibly models potential dependency of genetic sequence sampling on the population size. An added bonus of this new modeling framework is more precise estimation of population size changes. We demonstrate strengths of our new methodology on simulated data and on genetic sequences of Influenza and Ebola viruses.

## Introduction

Phylodynamic inference—the study and estimation of population dynamics from genetic sequences—relies upon data sampled in a timeframe compatible with the evolutionary dynamics under question [1]. One important class of phylodynamic methods seeks to estimate magnitudes and changes in a measure of genetic diversity called the *effective population size*, often considered proportional to the census population size [2] or number of infections in epidemiological contexts [3]. One subtle and often ignored complication of phylodynamic inference occurs when there is a probabilistic dependence between the effective population trajectory and the temporal frequency of collecting data samples, such as in case of sampling infectious disease agent genetic sequences with increasing urgency and intensity during a rising epidemic. This issue of *preferential sampling* was studied in depth by Karcher et al. in the limited context of a known, fixed genealogy reconstructed from the genetic data [4]. Karcher et al. demonstrated that conditioning on sampling times is not harmless when sampling protocols (implicitly) depend on effective population size. Such conditioning can be viewed as a model misspecification and results in biased estimation of the effective population size. Here, we extend the work of Karcher et al. and develop a Bayesian framework for accounting for preferential sampling during effective population size estimation directly from sequence data rather than from a fixed genealogy. We also propose a more flexible model for sequence sampling times that allows for inclusion of arbitrary time-dependent covariates and their interactions with the effective population size.

Methods for estimating effective population size from genealogical data and genetic sequence data have evolved from the earliest low dimensional parametric methods, such as constant population size [5] and exponential growth models [5, 6], to more flexible, nonparametric or highly parametric methods based on change-point models and Gaussian process smoothing [7, 8, 9, 10, 11, 12, 13]. Most coalescent-based methods condition on the times of sequence sampling, rather than include these times into the model, leaving open the possibility of model misspecification if preferential sampling over time is in play. This is in contrast to birth-death phylodynamic models that include the sequence sampling model by necessity [14, 15]. Volz and Frost and Karcher et al. introduced coalescent models that include sampling times as random variables, whose distribution is allowed to depend on the effective population size [15, 4]. In particular, Karcher et al. propose a method that models sampling times as an inhomogeneous Poisson process with log-intensity equal to an affine transformation of the log-transformed effective population size. In the presence of preferential sampling, this sampling-aware model demonstrates improved accuracy and precision compared to standard coalescent models due to eliminating an element of model misspecification and incorporating an additional source of information to estimate the effective population trajectory.

The main limitations of the approach in [4] are a reliance on a fixed, known genealogy and lack of flexibility in the preferential sampling time model that currently does not allow the relationship between effective population size and sampling intensity to change over time. We address the issue of fixed-tree inference by implementing a preferential sampling time model in the popular phylodynamic Markov chain Monte Carlo (MCMC) software package BEAST [16]. This allows us to perform inference directly from genetic sequence data, appropriately accounting for genealogical uncertainty, using a wide selection of molecular sequence evolution models and well tested phylogenetic MCMC transition kernels. Additionally, we implement a tuning parameter free elliptical slice sampling transition kernel [17] for high dimensional effective population size trajectory parameters, which allows us to update these parameters efficiently.

We also address the issue of an inflexible preferential sampling time model by incorporating time-varying covariates into the model. We model the sampling times as an inhomogeneous Poisson process with log-intensity equal to a linear combination of the log-effective population size and any number of functions of time. These functions can include time varying *covariates* and products of covariates and the log-effective population size, referred to as *interaction covariates*. The addition of covariates into the sampling time model allows for incorporating additional sources of information into the relationship between effective population size and sampling intensity. One example of time-varying covariates includes an exponential growth function to account for a continuous decrease in sequencing costs that results in increased intensity of genetic data collection over time. In the context of endemic infectious disease surveillance, it is likely important to account for seasonality when modeling changes in genetic data sampling intensity, motivating inclusion of periodic functions as time varying covariates in the preferential sampling model.

We validate our methods first by simulating genealogies and sequence data and confirming that our methods successfully reconstruct the true effective population trajectories and true model parameters. We briefly simulate data in a fixed-tree context to demonstrate the fundamentals of incorporating covariates into the sampling time model and what bias is introduced by model misspecifications. We proceed to simulate genetic sequence data and demonstrate that our model successfully functions when we estimate effective population size trajectory and other parameters directly from sequence data. We also use simulations to test a combination of the two extensions of the preferential sampling model and work with covariates while sampling over genealogies during the MCMC. Finally, we use our method to analyze two real-world epidemiological datasets. We analyze a USA/Canada regional influenza dataset [18] to determine if exponential growth of genetic sequencing or seasonal changes in sampling intensity are important to adjust for during effective population size reconstruction. We also analyze data from the recent Ebola outbreak in Western Africa to determine if preferential sampling has taken place and whether time-varying covariates or interaction covariates improve the phylodynamic inference.

## Methods

### Sequence data and substitution model

Consider an *alignment* **y** = {*y*_*ij*_}, *i* = 1, …, *n*, *j* = 1, …, *l*, of *n* genetic sequences across *l* sites, collected from a well-mixed population at *sampling times*

$$\begin{array}{c} s={\left\{s_{i}\right\}}_{i=1}^{n},\;s_{1}\geq \ldots \geq s_{n}=0. \end{array}$$

The following example shows an alignment of *n* = 5 samples across *l* = 10 sites, sampled at distinct times between time 7 and time 0—with time understood to be time *before* the latest sample:

$$\begin{array}{ccc} y_{1}= & ACATGAGCTT, & s_{1}=7\\ y_{2}= & ACTTGACCTG, & s_{2}=4\\ y_{3}= & TCTTGACCTT, & s_{3}=2\\ y_{4}= & AAATCTGCGT, & s_{4}=1\\ y_{5}= & AGATGTGCAT, & s_{5}=0. \end{array}$$

**Fig 1:**
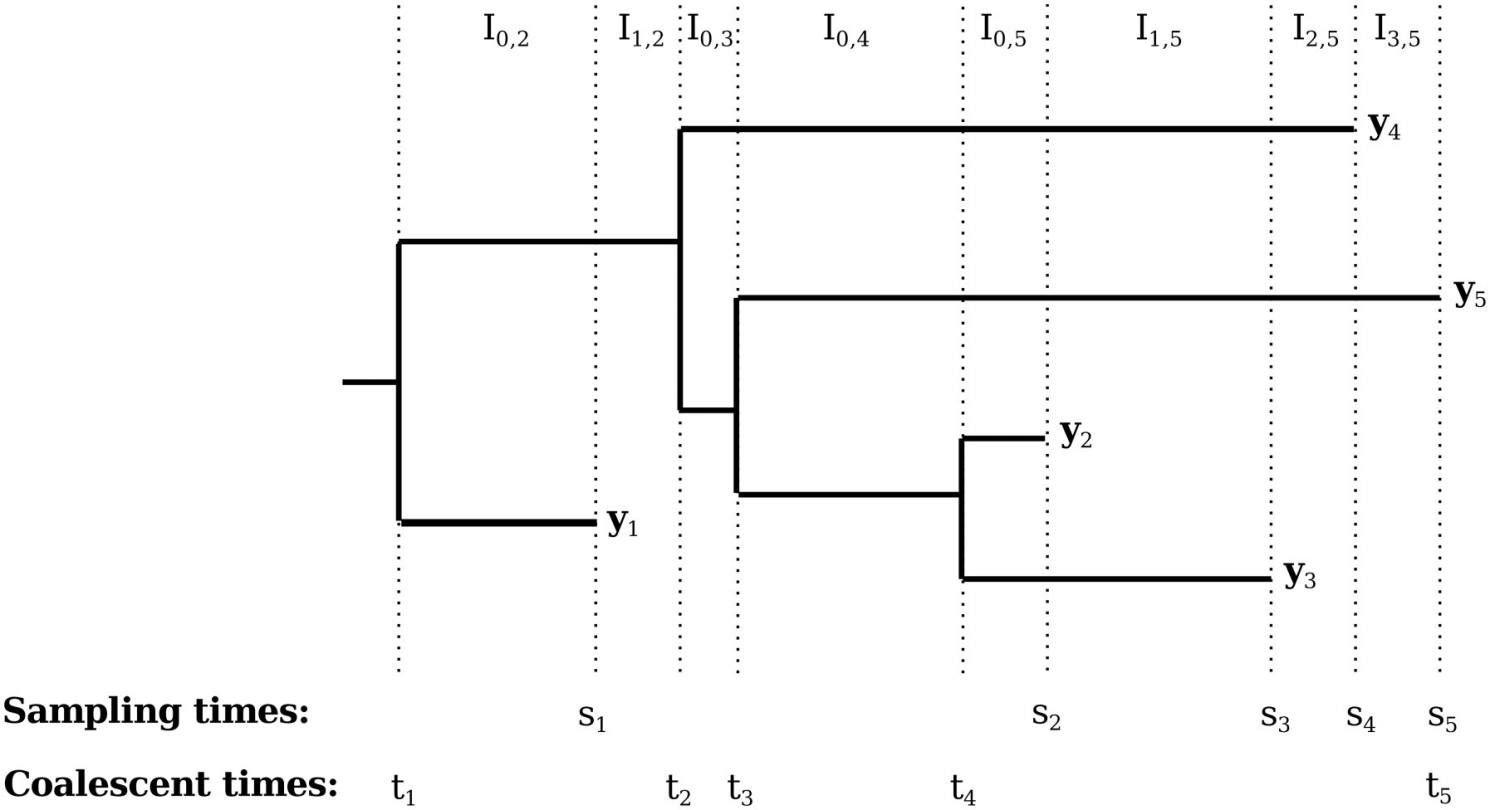
Illustration of an example heterochronous genealogy with *n* = 5 lineages. Sampling times *s*_1_, …, *s*_5_ and coalescent times *t*_1_, …, *t*_5_ are marked below the genealogy, and sequence data **y**_1_, …, **y**_5_ are marked at their corresponding tips.

All of the individual sequences share a common ancestry, which can be represented by a bifurcating tree called a *genealogy*—illustrated in Fig 1.

We assume that sequence data **y** are generated by a continuous time Markov chain (CTMC) *substitution model* that models the evolution of the genetic sequence along the genealogy **g**. According to this model, alignment sites are independent and identically distributed, with a transition matrix ***θ*** controlling the CTMC substitution rates between the different nucleotide bases. Some relaxation of these assumptions is possible [19]. Different substitution models are then defined by different parameterizations of ***θ*** [20]. It is simple to simulate from these models, and we can efficiently compute the probability of the observed sequence data **y**,

$$\begin{array}{c} \text{Pr}(y|g,\theta ), \end{array}$$

using Felsenstein’s pruning algorithm [21, 22].

### The coalescent

Recall that we assume that the *n* sampled sequences share a common ancestry, which can be represented by a bifurcating tree called a *genealogy*—illustrated in Fig 1. The branching events of the tree $g={\left\{t_{i}\right\}}_{i=1}^{n-1},t_{1}>\ldots >t_{n-1}$ (with *t* greater the farther *back* in time an event occurs) are called *coalescent events*. The times associated with the tips of the tree $s={\left\{s_{i}\right\}}_{i=1}^{n},s_{1}>\ldots >s_{n}$, are called *sampling times* or *sampling events*. If all of the sampling events are simultaneous, the sampling is called *isochronous*. Assuming that the population evolves according to the Wright-Fisher model of genetic drift and that the size of the population is not changing, [23] derived a probability density for an isochronous genealogy, where the population size plays the role of a parameter of this density. Since the Wright-Fisher model is a simplified representation of the evolutionary process, the above parameter is called the *effective population size*, *N*_*e*_. Later extensions to the coalescent model incorporated variable effective population size *N*_*e*_(*t*) [5] and the ability to evaluate densities of genealogies with *heterochronously* sampled tips—genealogies with non-simultaneous sampling times [24].

Given sampling times **s** and effective population size trajectory *N*_*e*_(*t*), we would like to define the probability density for a particular genealogy **g**. We use the term *active lineages*, *n*(*t*), to refer to the difference between the number of samples taken and the number of coalescent events occurred between times 0 and *t*. To illustrate, in Fig 1, *n*(*t*) can be seen as the number of horizontal lines that a vertical line at time *t* will cross. Suppose we partition the interval (*s*_*n*_, *t*_1_), from the most recent sampling event to the *time to most recent common ancestor* (TMRCA), into intervals *I*_*i*,*k*_ with constant numbers of active lineages. Let λc(t)=(n(t)2)/Ne(t). Then the coalescent density evaluated at genealogy **g** is

$$\begin{array}{c} \text{Pr}\left(g|N_{e}\left(t\right),s\right)\propto \prod_{k=2}^{n}[\lambda_{c}\left(t_{k-1}\right)\;\text{exp}\;(-\int_{I_{i,k}}\lambda_{c}\left(t\right)dt)]. \end{array}$$ (1)

### Population size prior

Note that without further assumptions the effective population size trajectory function *N*_*e*_(*t*) is infinite-dimensional, so inference about *N*_*e*_(*t*) without some manner of constraint is intractable. A number of approaches, reviewed in the Introduction, have been suggested to address this fact. Here, we take a regular grid approach that was used before in multiple studies [11, 12, 13, 4]. To review, we approximate *N*_*e*_(*t*) with a piecewise constant function, *N*_*γ*_(*t*) = exp[*γ*(*t*)], where $\gamma \left(t\right)=\sum_{i=1}^{p}\gamma_{i}1_{\left\{t\in J_{i}\right\}}$ and *J*_1_, …, *J*_*p*_ are consecutive time intervals of equal length. In contexts where the genealogy is known, we choose intervals that perfectly cover the interval between the TMRCA and the latest sample. However, in contexts where the genealogy is estimated from sequence data, the TMRCA is not necessarily fixed. To address this, we choose equal intervals that extend to a fixed point in time and append an additional interval that extends from that point infinitely back in time. This allows us to estimate the effective population trajectory with user-defined resolution over a window that extends back in time as far as the user chooses. The choice of the end point of the grid is up to the user, but it is advisable to choose a point that is farther back in time than an *a priori* estimate of the TMRCA in order to extend the high-resolution grid to cover the entire true genealogy.

The population size trajectory *N*_***γ***_(*t*) is parameterized by a potentially high dimensional vector ***γ*** = (*γ*_1_, …, *γ*_*p*_). We assume that *a priori* *γ* follows a first order Gaussian random walk prior with precision hyperparameter *κ*: $\gamma_{i}|\gamma_{i-1}∼N\left(\gamma_{i-1},1/\kappa \right)$ or, equivalently, that $\gamma_{i}-\gamma_{i-1}∼N\left(0,1/\kappa \right)$, for *i* = 2, …, *p*. We use a Gaussian prior on the first element: $\gamma_{1}∼N\left(0,\sigma_{p}^{2}\right)$. Finally, we assign a Gamma(*α*, *β*) hyperprior to *κ*.

### Preferential sampling model with covariates

 [4] model times at which sequences are collected as a Poisson point process with intensity λ_*s*_(*t*) equal to a log-linear function of the log effective population size. Although it is realistic to assume that the larger the population, the more members of the population gets sequenced, other factors may influence the distribution of sequence sampling times. For instance, decreasing sequencing costs may result in increasing sequence sampling intensity even if the population size remains constant. We propose an extension to the sampling model that allows for the incorporation of time-varying covariates as additional sources of information. Suppose we have one or more real-valued functions, $F=\{f_{2}\left(t\right),\ldots ,f_{m}\left(t\right)\}$. We let

$$\begin{array}{c} \text{log}\lambda_{s}\left(t;F\right)=\beta_{0}+\beta_{1}\gamma \left(t\right)+\beta_{2}f_{2}\left(t\right)+\ldots +\beta_{m}f_{m}\left(t\right)+[\delta_{2}f_{2}\left(t\right)+\ldots +\delta_{m}f_{m}\left(t\right)]\gamma \left(t\right), \end{array}$$ (2)

where we may set any or all of the *β*_2_, …, *β*_*m*_ or *δ*_2_, …, *δ*_*m*_ to zero if we want to avoid modeling effects of certain covariates or their interactions with the log-population size. Notice that we reserve *f*_1_(*t*) for *γ*(*t*) = log[*N*_*e*_(*t*)], which is the covariate that is always present in our model. We also point out that even though Eq (2) is written in continuous time, in practice we assume that both the sampling intensity λ_*s*_(*t*) and our time varying covariates are piecewise constant, with changes occurring at the grid points specified in Subsection. We assign independent $N(0,\sigma_{s}^{2})$ priors for all components of the preferential sampling model parameter vector ***β*** = (*β*_0_, *β*_1_, …, *β*_*m*_, *δ*_2_, …, *δ*_*m*_).

### Posterior approximation with MCMC

**Fig 2:**
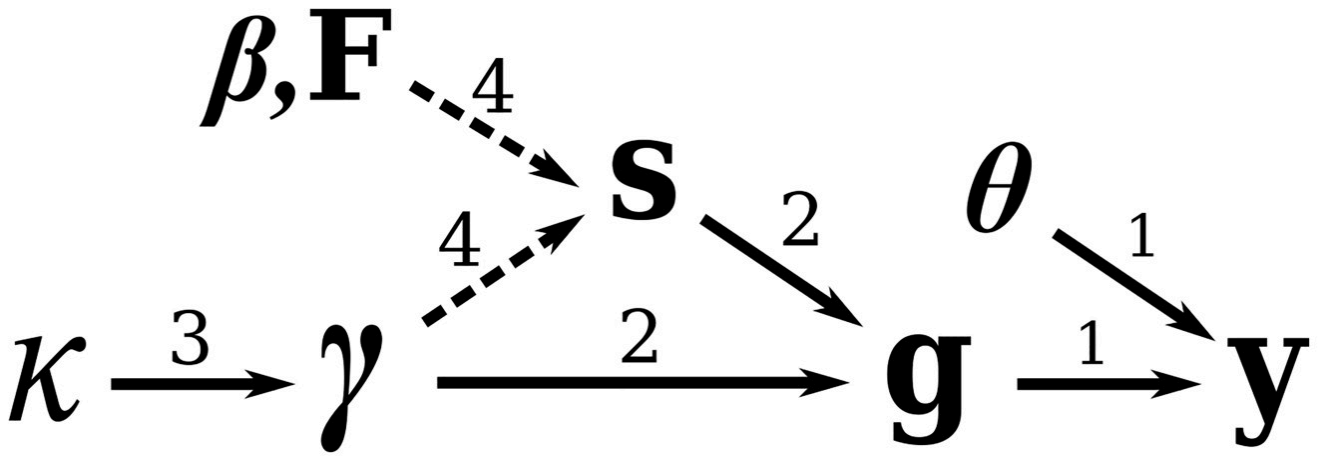
Dependency graph for the phylodynamic model parameters and data. Dependencies labeled 1 are explored in section, those labeled 2 are explored in section, those labeled 3 are explored in section, and those labeled 4 are explored in section. The dashed lines between γ,β,F and **s** represent preferential sampling.

Having specified all parts of our data generating model, we are now ready to define the posterior distribution of all unknown variables of interest:

$$\begin{array}{c} \begin{array}{cc} \text{Pr}(g,\gamma ,\kappa ,\beta ,\theta |y,s,F)\propto & \text{Pr}(y|g,\theta )\;\text{Pr}(g|\gamma ,s)\;\text{Pr}(s|\gamma ,\beta ,F)\;\text{Pr}(\gamma |\kappa )\\ & \times \text{Pr}(\kappa )\;\text{Pr}(\beta )\;\text{Pr}(\theta ), \end{array} \end{array}$$ (3)

where all probabilities and probability densities on the righthand side of Eq (3) are defined in the previous subsections. Fig 2 illustrates conditional dependencies of model parameters and data in a graph form.

When the distribution of sampling times does not depend on the effective population size trajectory (in our model, this happens when *β*_1_ = 0 and *δ*_2_ = ⋯ = *δ*_*m*_ = 0), the posterior takes the following form:

$$\begin{array}{c} \begin{array}{cc} \text{Pr}(g,\gamma ,\kappa ,\theta ,\beta |y,s,F) & \propto \underset{\propto \text{Pr}(g,\gamma ,\kappa ,\theta |y,s)}{\underset{︸}{\text{Pr}(y|g,\theta )\;\text{Pr}(g|\gamma ,s)\;\text{Pr}(\gamma |\kappa )\;\text{Pr}(\kappa )\;\text{Pr}(\theta )}}\\ & \times \underset{\propto \text{Pr}(\beta |s,F)}{\underset{︸}{\text{Pr}(s|\beta ,F)\;\text{Pr}(\beta )}}. \end{array} \end{array}$$

The factorization above demonstrates that when ***γ*** is absent from the Pr(**s**∣⋅) term, joint and separate estimations of effective population size parameters ***γ*** and preferential sampling model parameters ***θ*** will yield identical results. Moreover, in this case estimation of sampling model parameters can be dropped from the analysis entirely, since typically these parameters would be considered nuisance. If we drop preferential sampling, our model specifications reduces to the Bayesian skygrid model of [12], with the corresponding posterior:

$$\begin{array}{c} \text{Pr}(g,\gamma ,\kappa ,\theta |y,s)\propto \text{Pr}(y|g,\theta )\;\text{Pr}(g|\gamma ,s)\;\text{Pr}(\gamma |\kappa )\;\text{Pr}(\kappa )\;\text{Pr}(\theta ). \end{array}$$ (4)

We approximate posteriors (3) and (4) by devising MCMC algorithms, implemented in the software package BEAST [16], that target these distributions. We update model parameters in blocks—1) genealogy **g**, 2) substitution parameters ***θ***, 3) population size parameters ***γ***, 4) random walk prior precision *κ*, 5) preferential sampling model parameters ***β***—keeping parameters outside of the block fixed. We update the genealogy and substitution model parameters via the default BEAST Markov kernels. We update the log effective population size parameters ***γ*** via an elliptical slice sampler (ESS) operator [17, 25], which takes advantage of the Gaussian prior distribution of the latent field to perform efficient updates. Informally, it does this by sampling a set of parameter values from the prior and iteratively moving the values closer to the current values via elliptical interpolation if the coalescent likelihood falls below a random, but small, neighborhood of the current likelihood. Because the stepwise differences of the log effective population size trajectory, Δ***γ***, are modeled as independent Gaussians with precision *κ*, and because we give *κ* a Gamma(*α*, *β*) hyperprior, we update *κ* using a Normal-Gamma Gibbs update kernel with full conditional

$$\begin{array}{c} \kappa |\Delta \gamma ∼\text{Gamma}\;[\alpha +\frac{p}{2},\beta +\frac{1}{2}\sum_{i=2}^{p}{\left(\gamma_{i}-\gamma_{i-1}\right)}^{2}], \end{array}$$

where *p* is the number of parameters in the latent field. For our sampling conditional model with posterior (4), we finish here and refer to the method as *ESS/BEAST*, abbreviated when appropriate as *ESS*. For our sampling-aware model with the posterior (3), we update components of the preferential sampling model parameter vector ***β*** with univariate Gaussian random walk Metropolis-Hastings kernels. We refer to the method as *SampESS/BEAST*, abbreviated when appropriate as *SampESS*.

### Model selection and adequacy

To choose among preferential sampling models, we follow a standard Bayesian model selection approach by ranking these models according to their marginal likelihoods [26]. See Section B.1.5 in S1 Text for a quick review of marginal likelihood/Bayes factor-based model selection. Although this standard approach is helpful in choosing among a small number of candidate preferential sampling models, it does not answer an important question of whether any of these models are adequate enough to use instead of a more conventional conditional coalescent approach.

One way to probe model adequacy is posterior predictive model checking [27]. However, as we demonstrate in Sections B.1.3 and B.2.2 in S1 Text, posterior predictive model checking is often inconclusive with regard to the question of whether preferential sampling should be taken into account. So we propose a different strategy, where in addition to preferential sampling models, we use a model, where effective population size trajectory *N*_*e*_(*t*) and sampling rate λ(*t*) are unrelated and estimated nonparametrically using the same strategy that we use for nonparametric estimation of *N*_*e*_(*t*) in conditional and preferential sampling models. We call a preferential sampling model adequate if this model has higher marginal likelihood than the model with unrelated *N*_*e*_(*t*) and λ(*t*). For completeness, we also include into our marginal likelihood ranking a model that explicitly assumes uniform sampling of sequences across time. Currently, we implemented the unrelated *N*_*e*_(*t*) and λ(*t*) model and its marginal likelihood calculation using INLA for a fixed tree. We plan to add this model to BEAST in the future so we can take advantage of multiple robust marginal likelihood calculation procedures that are already implemented in BEAST [28].

### Implementation

We implemented INLA-based, fixed-genealogy BNPR-PS method with simple covariates in R package phylodyn (https://github.com/mdkarcher/phylodyn). The package has also MCMC functionality that can handle inference from a fixed genealogy with simple and interaction sampling model covariates. See phylodyn vignettes for more details. MCMC for direct inference from sequence data is available in the development branch of software package BEAST (https://github.com/beast-dev/beast-mcmc). We provide examples of how to specify our preferential sampling models in BEAST xml files at https://github.com/mdkarcher/BEAST-XML. The last repository also contains XML files needed to replicate our simulations and real data analyses.

## Results

### Simulation study

#### Inference assuming fixed genealogy

In Section, we proposed an extended sampling time model that incorporated time-varying covariates. We perform a simulation study to confirm the ability of our method to recover the true effective population trajectory and model coefficients with covariates affecting the sampling intensity. We begin here with fixed genealogies and move on to direct inference from sequence data in the next section.

We start with the inhomogeneous Poisson process sampling model with log-intensity as in Eq 2. If we restrict all *β*s and *δ*s to be zero aside from *β*_0_, the model collapses to homogenous Poisson process sampling (equivalently, uniformly sampling a Poisson number of points across the sampling interval). If we allow *β*_1_ to be nonzero, the model becomes the sampling-aware model in [4]. If we allow additional *β*s, each corresponding to a fixed function of time, to be nonzero (but not *δ*s) we say that the model includes *simple* or *ordinary covariates*.

For computational efficiency in this simulation study, we build upon the methods from [4], including Bayesian Nonparametric Population Reconstruction (BNPR) which uses integrated nested Laplace approximation (INLA) to efficiently approximate the marginal posterior for fixed-genealogy data, and Bayesian Nonparametric Population Reconstruction with Preferential Sampling (BNPR-PS) which does the same but includes our sampling time model (without covariates). We incorporate our extended sampling time model into BNPR-PS, but due to constraints in the INLA R package, upon which BNPR-PS relies, we can only include simple covariates.

Because our sampling time model is an inhomogeneous Poisson process, it is straightforward to simulate sampling times. We use a *time-transformation* method [29, pages 98–99], which, informally, treats the waiting times between events as transformations of exponential waiting times based on the intensity function following the previous event. Because the coalescent likelihood is sufficiently similar to an inhomogeneous Poisson process, we can use a similar time-transformation technique to generate the coalescent events of simulated genealogies [30]. We implement these methods for simulating sampling times and coalescent times in R package phylodyn [31].

**Fig 3:**
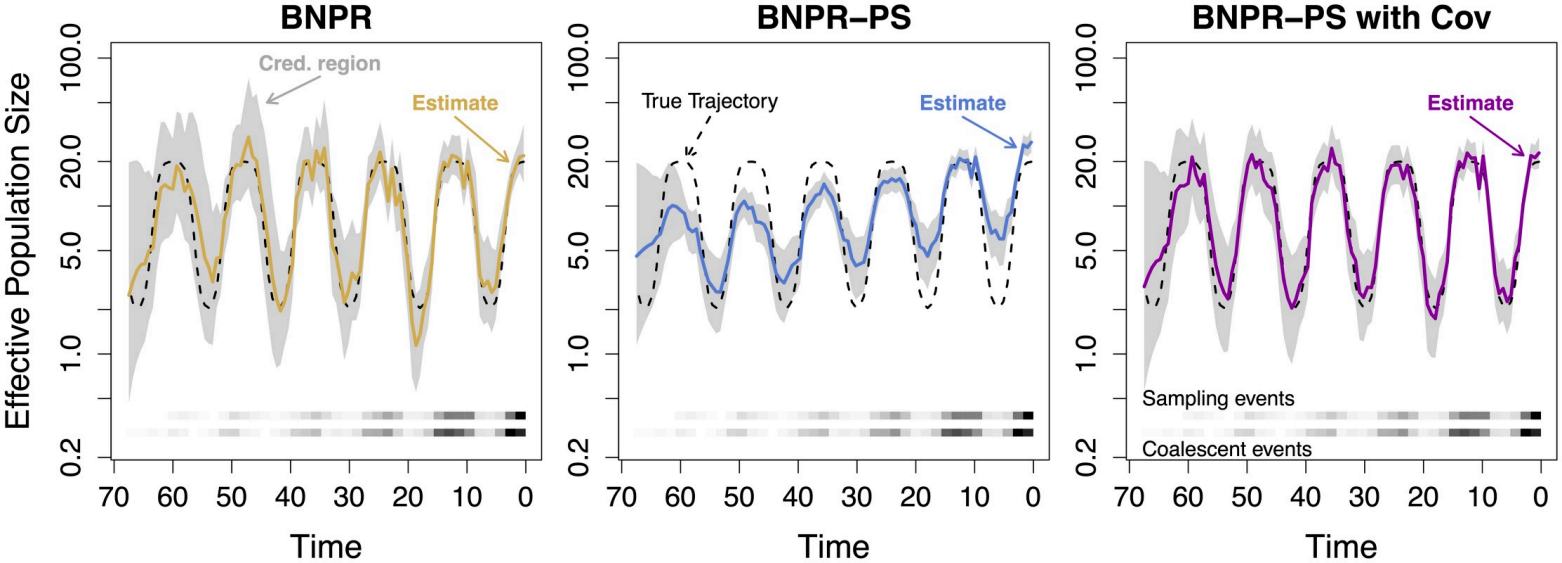
Effective population size reconstruction for BNPR, BNPR-PS, and BNPR-PS with simple covariates. The dotted black line represents the true effective population trajectory. The solid colored line represents the marginal posterior median effective population trajectory inferred by BNPR (yellow), BNPR-PS (blue), and BNPR-PS with simple covariates (purple), and the gray region represents the corresponding pointwise 95% credible intervals for the effective population trajectory. The log sampling intensity was 1.557 + *γ*(*t*) − 0.025*t*.

In Fig 3, we illustrate BNPR, BNPR-PS, and BNPR-PS with simple covariates applied to a single simulated genealogy with sampling events distributed according to log-intensity 1.56 + *γ*(*t*) − 0.05*t*, resulting in 1013 tips, where *γ*(*t*) = log[*N*_*e*,2,6_(*t*)] and *N*_*e*,*a*,*o*_(*t*) is a family of functions that approximate seasonal changes in effective population size, defined as follows:

$$\begin{array}{c} N_{e,a,o}\left(t\right)=\{\begin{array}{cc} 2+18/(1+\text{exp}\{a[3-(t+o\;\text{(mod}\;\text{12)})]\}), & \text{if}\;t+o\;\text{(mod}\;\text{12)}\;\leq 6,\\ 2+18/(1+\text{exp}\{a[3+(t+o\;\text{(mod}\;\text{12)})-12]\}), & \text{if}\;t+o\;\text{(mod}\;\text{12)}\;>6. \end{array} \end{array}$$ (5)

We see that BNPR (the sampling conditional model) suffers from the kind of model misspecification induced bias illustrated in [4]. BNPR-PS with no additional covariates beyond *γ*(*t*) = log[*N*_*e*_(*t*)], in contrast, suffers even more strongly from a misspecified sampling model. Table 1 shows that the model fails to correctly infer the coefficient of *γ*(*t*). This illustrates the care one must take in choosing parameterizations of the sampling model. BNPR-PS with simple covariates, *γ*(*t*) and −*t*, the correctly-specified model, produces a reconstruction of the effective population trajectory that is very close to the true trajectory used to simulate the data. Table 1 shows that the true values of the sampling model coefficients are within 95% Bayesian credible intervals produced by our inference method with the correctly specified model.

**Table 1:** Summary of simulated fixed-tree data inference. Posterior distribution quantile summaries for BNPR-PS with no covariates (model: {*γ*(*t*)}, first row) and BNPR-PS with an ordinary covariate (model: {*γ*(*t*), −*t*}, second and third rows).

Model	Coef	Q0.025	Median	Q0.975	Truth
{*γ*(*t*)}	*γ*(*t*)	1.67	1.99	2.34	1.0
{*γ*(*t*), −*t*}	*γ*(*t*)	0.86	1.01	1.16	1.0
	−*t*	0.040	0.047	0.053	0.050

We also perform a simulation study, where we repeatedly simulate a genealogy from a fixed effective population size trajectory and use this genealogy to perform Bayesian inference. Without model misspecification, frequentist properties of our Bayesian estimators improve when when the number of sampled sequences/lineages increases, with bias diminishing and coverage of credible intervals converging to the nominal level. In addition to validating our method on simulations with no model misspecification, we investigate behavior of our Bayesian inferential procedure in situations when the sampling model is misspecified. As expected, such model misspecification, which includes ignoring preferential sampling when it is present, results in systematic bias, underscoring the importance of scrutinizing preferential sampling model adequacy. See Section B.2.1 in S1 Text for more details.

#### Model selection and adequacy assuming fixed genealogy

We test our model selection and checking model adequacy strategies using the same simulated data that we use in Section. We observe frequency of our marginal likelihood ranking procedure selecting the correct model approaches 1.0, as we increase the number of sampled sequences/lineages. More importantly, we find that when effective population size and sampling intensity are not related in any way, the marginal likelihood ranking frequently selects the unrelated *N*_*e*_(*t*) and λ(*t*) model, which correctly points to the conclusion that none of the preferential sampling models under consideration is adequate and it is best to stick with the standard conditional coalescent model. See Section B.2.1 in S1 Text for more details.

#### Direct inference from sequence data

We simulate several genealogies and DNA sequences from different sampling scenarios in order to evaluate how well our population reconstruction and parameter inference performs. Given a sampling model and, optionally, an effective population size trajectory, we generate sampling times within a *sampling window*. We generate sampling and coalescent times for a genealogy using the same time-transformation methods as for our fixed-tree simulations. We simulate the topology of the genealogy by proceeding backward in time, adding an active lineage at each sampling time and joining a pair of active lineages uniformly at random at each coalescent event. We provide an implementation of this tree-topology simulation method in phylodyn. We generate simulated sequence alignments using the software SeqGen [32], using the Jukes-Cantor 1969 (JC69) [33] substitution model. Initially, we set the substitution rate to produce an expected 0.9 substitutions per site, in order to produce a sequence alignment with many sites having one mutation, and some sites having zero or multiple mutations. As we discuss below, we also experiment with the substitution rate of 0.09 substitutions per site in a subset of our simulations. For all of our simulations, we use the same seasonal effective population trajectory, *N*_*e*,2,6_(*t*), as for our fixed-tree simulations.

**Fig 4:**
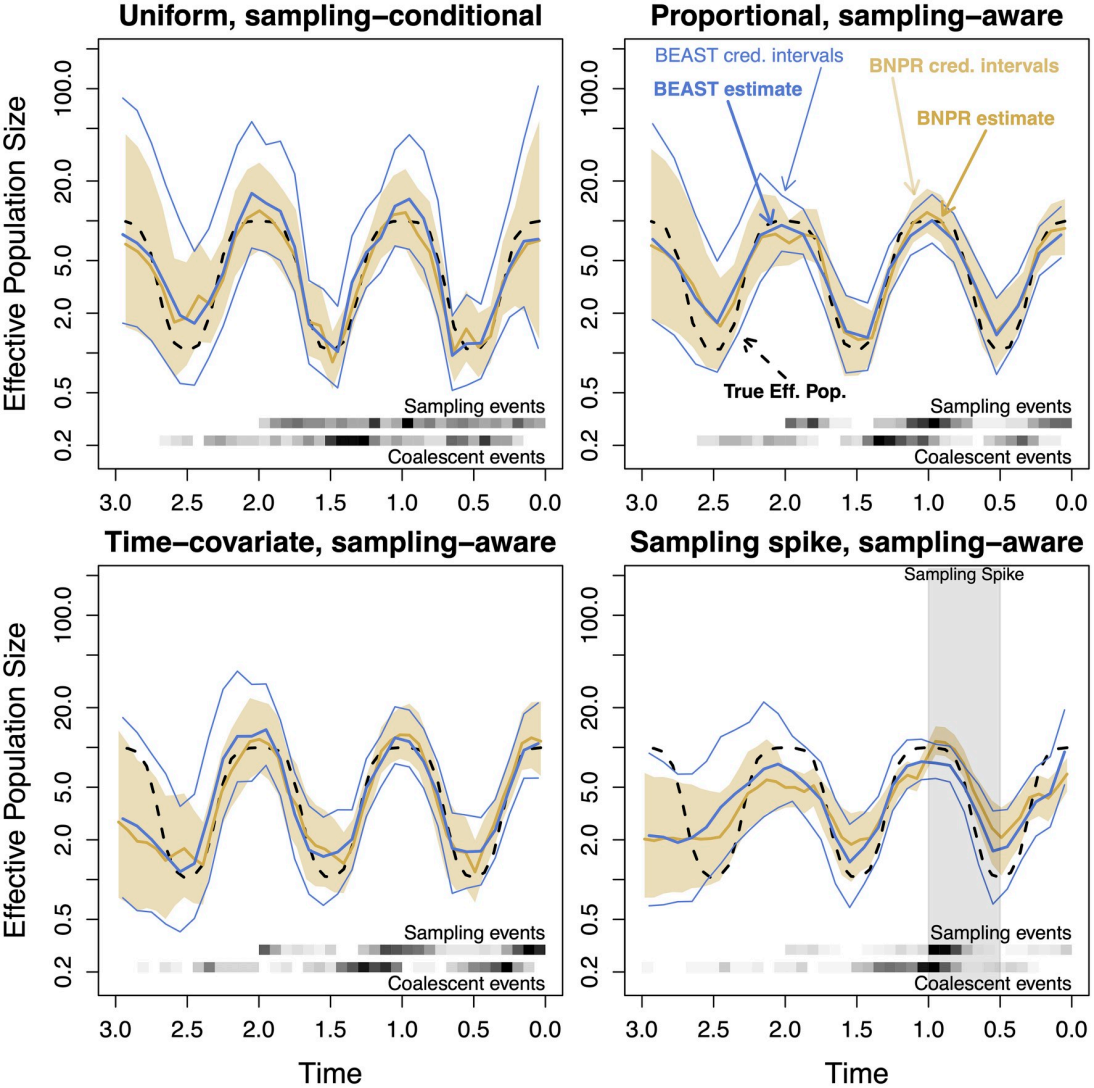
Effective population size reconstructions for four sequence data simulations, all based on the same seasonal effective population size trajectory. *Upper left*: Uniform sampling times, sampling-conditional posterior. *Upper right*: Sampling frequency proportional to effective population size, sampling-aware posterior. *Lower left*: Sampling frequency proportional to effective population times a time-covariate (exp(*t*)), sampling- and covariate-aware posterior. *Lower right*: Sampling frequency proportional to effective population size with a sampling spike, sampling- and covariate-aware posterior.

First, we simulate a genealogy with 200 tips and sequence data with 1500 sites and uniform sampling times and apply both of our sampling-conditional methods. We apply the INLA-based fixed-tree BNPR from [4] to the true genealogy, and we apply the MCMC-based tree-sampling ESS/BEAST (specified above) to the sequence data. In Fig 4 (upper left), we compare the truth with the resulting pointwise posterior medians and credible intervals. The two methods’ results are mutually consistent, with additional uncertainty in the tree-sampling method (visible in the wider credible intervals) due to having to estimate the genealogy jointly with other model parameters. We see similar results comparing BNPR-PS with SampESS/BEAST in Fig 4 (upper right), where we sample sequences (1500 sites) with sampling times generated from an inhomogeneous Poisson process with intensity proportional to effective population size (log-intensity 2.9 + *γ*(*t*)) resulting in 170 samples and infer using a sampling model with log-intensity *β*_0_ + *β*_1_*γ*(*t*). We also see similar results in Fig 4 (lower left), where we add time as an additional covariate and sample sequences (1500 sites) with log-intensity 3.35 + *γ*(*t*) − 0.5*t*, resulting in 199 samples, and perform inference using a sampling model with log-intensity *β*_0_ + *β*_1_*γ*(*t*) + *β*_2_ ⋅ (−*t*). Table 2 shows that SampESS does a reasonable job at reconstructing the true model coefficients, though the credible interval for −*t* includes 0.

**Table 2:** Summary of simulated sequence data inference. Posterior distribution quantile summaries for BEAST implementation of BNPR-PS models: with no covariates (model: {*γ*(*t*)}, first row), with an ordinary covariate (model: {*γ*(*t*), −*t*}, second and third row), with both an ordinary and interaction covariate (model: {*γ*(*t*), 1_*t*∈[0.5,1]_, 1_*t*∈[0.5,1]_ ⋅ *γ*(*t*)}, last three rows).

Model	Coef	Q0.025	Median	Q0.975	Truth
{*γ*(*t*)}	*γ*(*t*)	0.98	1.42	2.16	1.0
{*γ*(*t*), −*t*}	*γ*(*t*)	0.75	1.06	1.55	1.0
	−*t*	-0.06	0.44	0.94	0.5
{*γ*(*t*), 1_*t*∈[0.5,1]_, 1_*t*∈[0.5,1]_ ⋅ *γ*(*t*)}	*γ*(*t*)	0.72	1.26	2.14	1.0
	1_*t*∈[0.5,1]_	-9.01	-1.50	1.64	0.0
	1_*t*∈[0.5,1]_ ⋅ *γ*(*t*)	0.13	1.75	5.75	1.0

We also simulate a genealogy and sequence data (1500 sites) with log-intensity 1.89 + *γ*(*t*) + *γ*(*t*) ⋅ 1_*t*∈[0.5,1]_, resulting in 210 samples. This produces an interval we refer to as a *sampling spike* which requires the use of an interaction covariate. Because of design limitations of the R implementation of INLA, we are limited in how we may implement interaction covariates in BNPR-PS. Therefore, in Fig 4 (lower right) we plot SampESS/BEAST with the correct interaction covariate (and a corresponding ordinary covariate) against BNPR-PS with no covariates. We see SampESS (with covariates) perform better than BNPR-PS (without covariates) at reconstructing the correct trajectory. We also see that our method, using the full covariate model, with log-intensity *β*_0_ + *β*_1_*γ*(*t*) + *β*_2_ ⋅ 1_*t*∈[0.5,1]_ + *δ*_2_ ⋅ *γ*(*t*) ⋅ 1_*t*∈[0.5,1]_, produces a 95% Bayesian credible interval for the coefficient of the ordinary covariate that contains the true value (*β*_2_ = 0), while the true value of the interaction covariate coefficient (*δ*_2_ = 1) is correctly inside the 95% Bayesian credible interval produced by SampESS/BEAST.

We repeat simulations with proportional preferential sampling without covariates and with a simple time covariate 10 times and show the corresponding population size reconstructions in Figures B-8 and B-9 in S1 Text. In addition, we repeat proportional preferential sampling without covariates and with a simple time covariate simulations 10 more times, while reducing the substitution rate from 0.9 to 0.09 (Figures B-10 and B-11 in S1 Text). Both high (0.9) and medium (0.09) substitutions rate simulations pass a sanity check: most of the effective population size reconstructions recover the true trajectory and show wider credible intervals when we increase the phylogenetic uncertainty in the medium substitution rate regime.

### Seasonal influenza example

**Fig 5:**
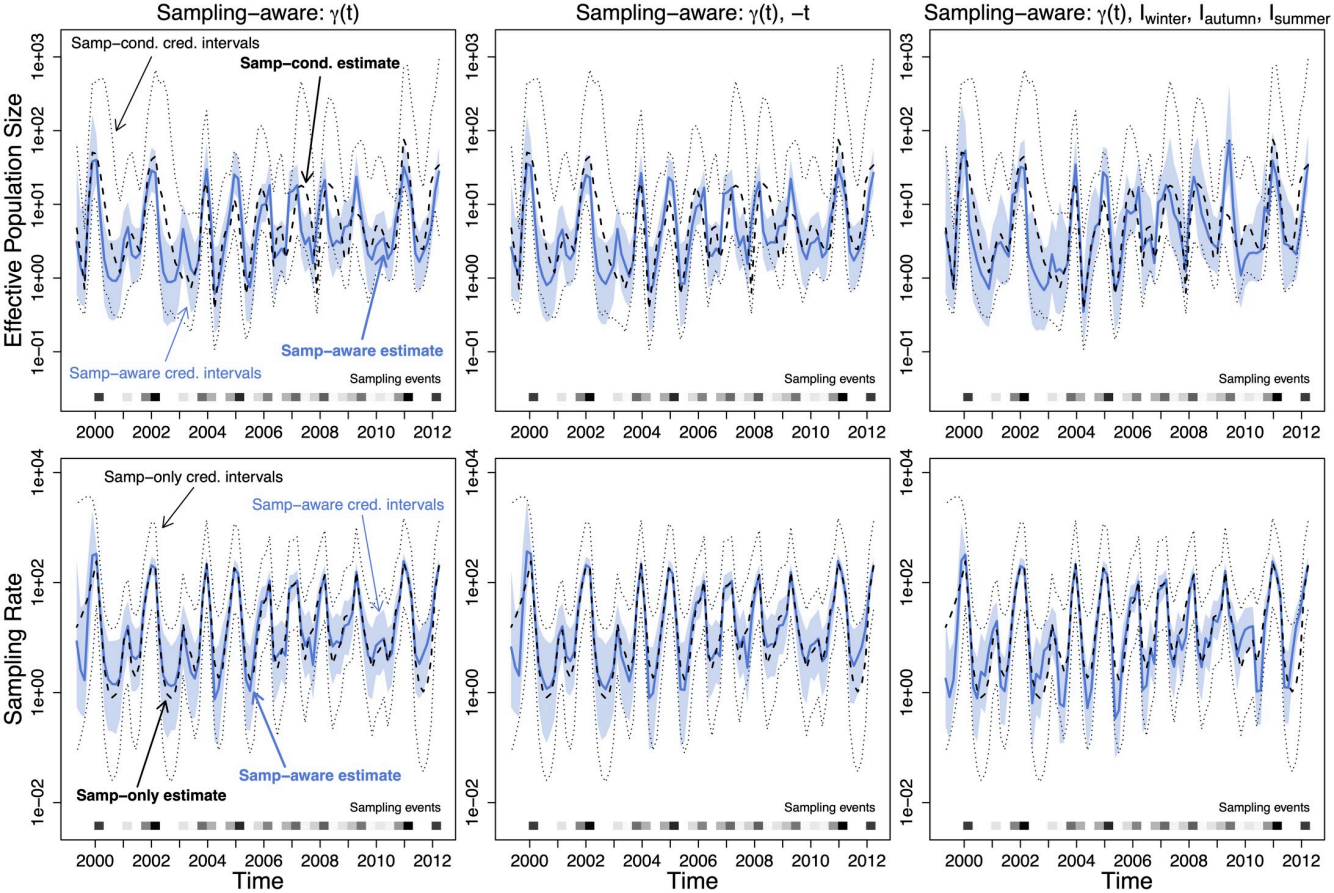
Effective population size and sampling rate reconstructions for the USA and Canada influenza dataset. *Upper row*: Dashed lines and dotted black lines are the pointwise posterior effective population size estimates and credible intervals of the sampling-conditional model. The blue lines and the light blue regions are the pointwise posterior effective population size estimates and credible intervals of that column’s sampling-aware model. *Lower row*: Dashed lines and dotted black lines are the pointwise posterior sampling rate estimates and credible intervals of a nonparametric sampling-time-only model. The blue lines and the light blue regions are the pointwise posterior sampling rate estimates and credible intervals of that column’s sampling-aware model.

We reanalyze the H3N2 regional influenza data for the USA/Canada region as analyzed with fixed-tree methods in [4]. The data contain 520 sequences aligned to form a multiple sequence alignment with 1698 sites of the hemagglutinin gene. This dataset is a subset of the dataset of influenza sequences from around the world analyzed in [18]. We use ESS/BEAST with our tree-sampling MCMC targeting posterior (4) to analyze these data and mark the pointwise posterior median and 95% credible region in black, summarized in Fig 5 (upper row). We observe a seasonal pattern consistent with flu seasons observed in the temperate northern hemisphere [18]. Our results are also consistent with previous fixed-tree method results but with larger credible interval widths due to correctly accounting for genealogical uncertainty in our analysis.

We apply our sampling-aware model SampESS/BEAST to the USA/Canada influenza data, following the posterior from Eq (3). We used several different log-sampling-intensity models. The simplest one has log-intensity *β*_0_ + *β*_1_*γ*(*t*) (abbreviated {*γ*(*t*)}) and is summarized in Fig 5 (upper left). We include a time term in one model, with log-intensity *β*_0_ + *β*_1_*γ*(*t*) + *β*_2_ ⋅ (−*t*) (abbreviated {*γ*(*t*), −*t*}) summarized in Fig 5 (upper center). We use seasonal indicator functions in the final model, defined as,

$$\begin{array}{cc} I_{\text{winter}}\left(t\right) & =I_{\left(t\;\text{mod}\;1.0)\in [0,0.25\right)},\\ I_{\text{autumn}}\left(t\right) & =I_{\left(t\;\text{mod}\;1.0)\in [0.25,0.5\right)},\\ I_{\text{summer}}\left(t\right) & =I_{\left(t\;\text{mod}\;1.0)\in [0.5,075\right)}, \end{array}$$

with *t* measured in decimal calendar years (going forward in time). This results in the log-intensity *β*_0_ + *β*_1_*γ*(*t*) + *β*_2_*I*_winter_(*t*) + *β*_3_*I*_autumn_(*t*) + *β*_4_*I*_summer_(*t*) (abbreviated {*γ*(*t*), *I*_winter_, *I*_autumn_, *I*_summer_}), summarized in Fig 5 (upper right).

We summarize the sampling model coefficient results for each model in Table 3. The {*γ*(*t*)} model corresponds to the preferential sampling model of [4], but has noticeably different estimates. We attribute this to the differences between the fixed-tree (with a tree inferred using a constant effective population size BEAST model), INLA-based approach of [4], and the tree-sampling MCMC-based approach of this paper. We also note that the {*γ*(*t*), −*t*} model does not perform better (or even noticeably differently) than the {*γ*(*t*)} model. The coefficient summary for {*γ*(*t*), −*t*} bears this out, because the 95% Bayesian credible interval for the coefficient for −*t* contains 0. This is expected as each year has approximately the same number of sequences, so there should be no exponential growth of sampling intensity. We do observe differences in the {*γ*(*t*), *I*_winter_, *I*_autumn_, *I*_summer_} model. The coefficient of *γ*(*t*) is close to 1.0, which is the easiest value to interpret under preferential sampling, suggesting a baseline sampling rate proportional to effective population size. The coefficients for the indicators suggest increased sampling in the flu season intervals, as compared to the summer intervals and especially the spring intervals—with spring treated as a baseline rate without an indicator for the sake of identifiability. We also fit a model with seasonal indicator covariates and their interactions with the log-effective population size, but do not find any support for including the interaction covariates into the preferential sampling model (see Section A.1 in S1 Text).

**Table 3:** Summary of USA/Canada influenza data inference. Posterior distribution quantile summaries for BEAST implementation of BNPR-PS models: with no covariates (model: {*γ*(*t*)}), with an ordinary covariate (model: {*γ*(*t*), −*t*}), and with seasonal indicator covariates (model: {*γ*(*t*), *I*_winter_, *I*_autumn_, *I*_summer_}). Using a fixed tree, we also include marginal likelihoods (MLs) for all of the above models, plus a model with *N*_*e*_(*t*) and λ_*s*_(*t*) estimated independently and nonparametrically (Unrelated *N*_*e*_(*t*) and λ_*s*_(*t*)) and a conditional model combined with a constant sampling intensity model for sampling times (Constant λ_*s*_(*t*)).

Model	ML	Coef	Q0.025	Median	Q0.975
Unrelated *N*_*e*_(*t*) and λ_*s*_(*t*)	-732	—	—	—	—
Constant λ_*s*_(*t*)	-975	—	—	—	—
{*γ*(*t*)}	-626	*γ*(*t*)	1.11	1.45	2.01
{*γ*(*t*), −*t*}	-632	*γ*(*t*)	1.21	1.52	2.00
		−*t*	-0.10	-0.02	0.07
{*γ*(*t*), *I*_winter_, *I*_autumn_, *I*_summer_}	-604	*γ*(*t*)	0.72	0.92	1.21
		*I*_winter_	1.91	2.79	3.83
		*I*_autumn_	1.88	2.85	3.85
		*I*_summer_	0.44	1.52	2.58

**Fig 6:**
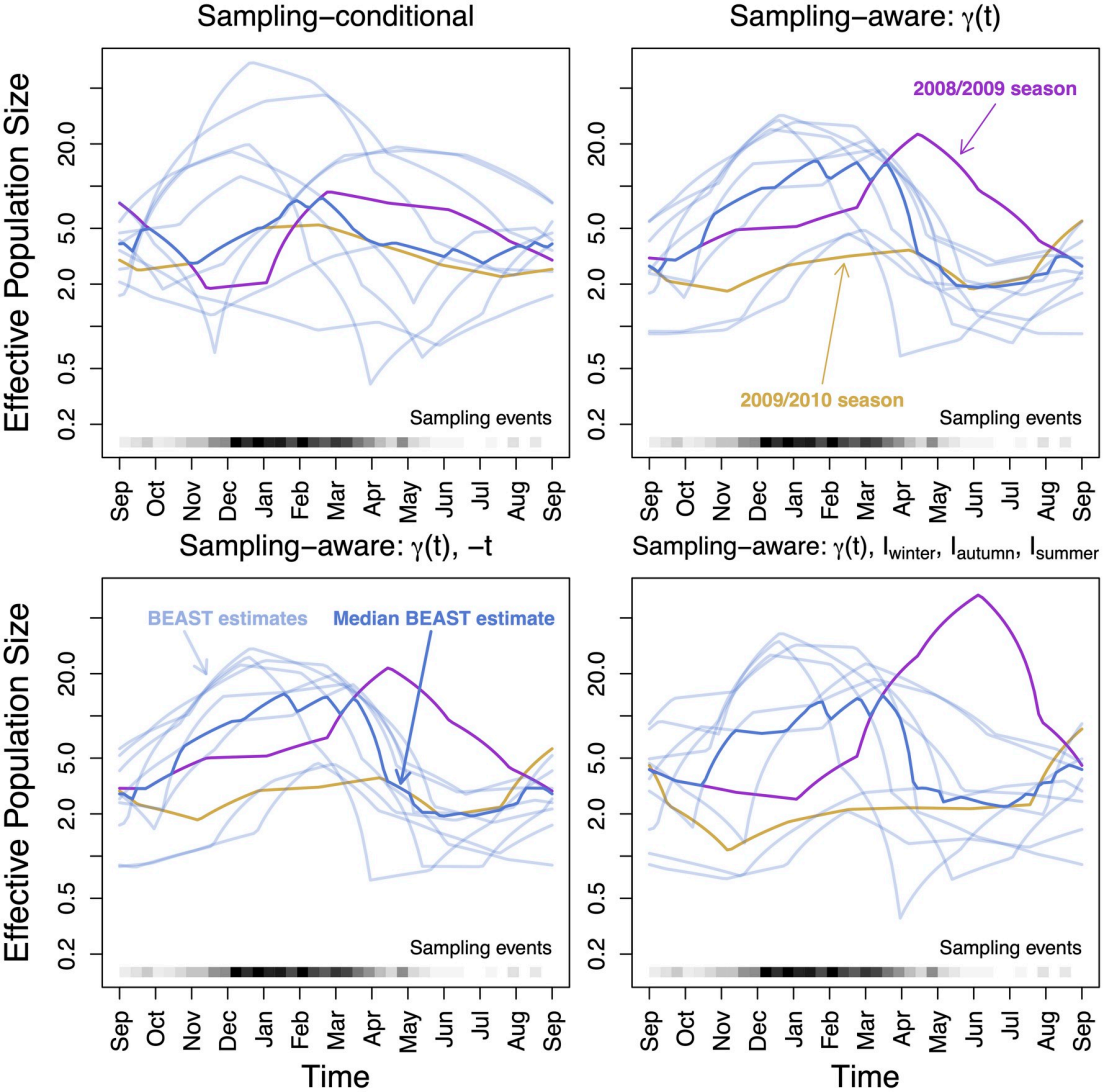
Effective population size seasonal overlay for the USA and Canada influenza dataset. The light blue lines are the pointwise posterior estimates for each year, and the dark blue line is the median annual estimate. *Upper left*: Sampling-conditional posterior. *Upper right*: Sampling-aware posterior with only log-effective population size *γ*(*t*) informing the sampling time model. *Lower left*: Sampling- and covariate-aware posterior, with *γ*(*t*) and −*t*. *Lower right*: Sampling- and covariate-aware posterior, with *γ*(*t*) and seasonal indicators *I*_winter_, *I*_autumn_, *I*_summer_.

We observe the seasonality of our estimates of the effective population size trajectory. In Fig 6, we superimpose the twelve years of estimates per model, and plot the posterior median annual estimate. We note that the sampling aware models all show increased seasonality compared to the sampling conditional model. We also note that the 2008-2009 flu season stands out on the seasonality plot for having a peak in the summer months of 2009, particularly in the preferential sampling models. This behavior is most likely due to a misspecification of our model for sampling intensity. This misspecification is expected given the first documented emergence of the H1N1 strain in the United States in April of 2009 and the resulting, unaccounted in our model, increased surveillance of all influenza strains in summer 2009 [34]. Higher than usual sampling intensity in summer 2009 makes our preferential sampling models conclude that the effective population size during this time period must be also elevated. Also, note that the estimated effective population size of H3N2 strain during 2009/2010 flu season is markedly lower than during most of other seasons. This is in line with the H1N1 strain successfully competing with the H3N2 strain, resulting in the lower prevalence of the latter.

To check adequacy of the preferential sampling models, we compare the posterior distributions of sampling intensities obtained via our BEAST implementation of BNPR-PS with a nonparametric INLA-based estimate of the sampling rate (using a method similar to BNPR-PS without the coalescent likelihood or covariates). Fig 5 (lower row) shows the comparison. The methods produce very similar estimates, with the BEAST/MCMC methods having thinner credible intervals due to incorporating additional information from the coalescent likelihood. However, when we apply our model selection strategy to a fixed majority clade credibility phylogenetic tree used in [4], the preferential sampling model with seasonal covariates emerges as a winner with the highest marginal likelihood (see Table 3). We note that all but constant λ_*s*_(*t*) preferential sampling models outperform the unrelated *N*_*e*_(*t*) and λ_*s*_(*t*) model. We also performed posterior predictive checking, described in Section B.1 in S1 Text, but found that this method lacked power to discriminate among preferential sampling models (see Section B.5.1 in S1 Text).

### Ebola outbreak

Next, we analyze a subset of the Ebola virus sequences arising from the recent Western Africa Ebola outbreak (as collated in [35]). The data consist of 1610 aligned whole genomes, collected from mid-2014 to mid-2015. The resulting alignment has 18,992 sites. The dataset represents over 5% of known cases of Ebola detected during that outbreak, providing an unprecedented insight into the epidemiological dynamics of an Ebola outbreak. We consider two subsets of the data, corresponding to the samples from Sierra Leone and Liberia. For Sierra Leone, we subsampled 200 sequences, chosen uniformly at random out of 1010 samples for computational tractability. For Liberia, we use the entire collection of 205 sequences obtained from infected individuals in this country.

**Fig 7:**
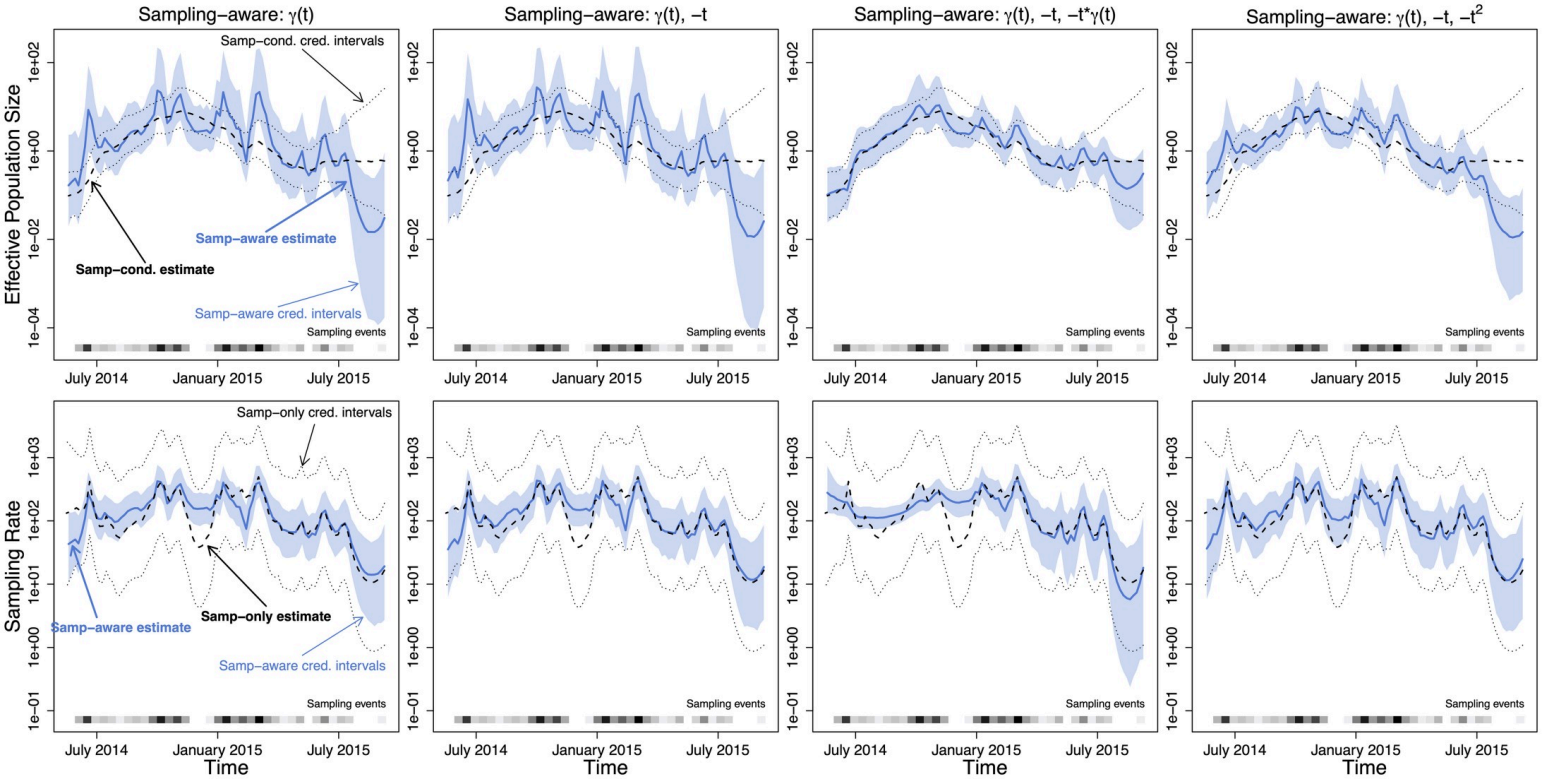
Effective population size and sampling rate reconstructions for the Sierra Leone Ebola dataset. *Upper row*: Dashed lines and dotted black lines are the pointwise posterior effective population size estimates and credible intervals of the sampling-conditional model. The blue lines and the light blue regions are the pointwise posterior effective population size estimates and credible intervals of that column’s sampling-aware model. *Lower row*: Dashed lines and dotted black lines are the pointwise posterior sampling rate estimates and credible intervals of a nonparametric sampling-time-only model. The blue lines and the light blue regions are the pointwise posterior sampling rate estimates and credible intervals of that column’s sampling-aware model.

We begin by applying ESS/BEAST method with no preferential sampling to the Sierra Leone dataset. We use MCMC to target our tree-sampling posterior from Eq (4) and depict the pointwise posterior median effective population curve, *N*_*e*_(*t*), with a black dashed line and its corresponding 95% credible region boundaries with black doted lines, shown in all panels of the first row of Fig 7. The resulting effective population size trajectory visually resembles a typical epidemic trajectory of prevalence or incidence that peaks in Autumn of 2014. Next, we apply our sampling-aware model SampESS/BEAST to the Ebola data, targeting with MCMC the posterior from Eq (3). We use several different log-sampling-intensity models. The simplest model, abbreviated as {*γ*(*t*)}, has log-sampling-intensity *β*_0_ + *β*_1_*γ*(*t*). We include a *t* term in our next sampling model, abbreviated as {*γ*(*t*), −*t*}, with log-intensity *β*_0_ + *β*_1_*γ*(*t*) + *β*_2_ ⋅ (−*t*). This model postulates that even if the effective population size remains constant, the sampling intensity is growing or declining exponentially. We make −*t* an interaction covariate as well in the next model, abbreviated {*γ*(*t*), −*t*, −*t* ⋅ *γ*(*t*)}, resulting in the log-sampling-intensity *β*_0_ + *β*_1_*γ*(*t*) + *β*_2_ ⋅ (−*t*) + *δ*_2_ *γ*(*t*) ⋅ (−*t*). For the final model, we include −*t* and −*t*^2^ as ordinary covariates, abbreviated {*γ*(*t*), −*t*, −*t*^2^}, with log-sampling-intensity *β*_0_ + *β*_1_*γ*(*t*) + *β*_2_ ⋅ (−*t*) + *β*_3_ ⋅ (−*t*^2^). The resulting posterior distribution summaries of the effective population size trajectory are shown in blue in the upper row of Fig 7. Parameter estimates of all models fit to the Sierra Leone data are summarized in Table 4.

**Table 4:** Summary of Sierra Leone Ebola sequence data inference. Posterior distribution quantile summaries for BEAST implementation of BNPR-PS models: with no covariates (model: {*γ*(*t*)}), with an ordinary covariate (model: {*γ*(*t*), −*t*}), with both an ordinary and interaction covariate (model: {*γ*(*t*), −*t*, −*t*⋅*γ*(*t*)}), and with linear and quadratic ordinary covariates (model: {*γ*(*t*), −*t*, −*t*^2^}). Using a fixed tree, we also include marginal likelihoods (MLs) for all of the above models with no interactions, plus a model with *N*_*e*_(*t*) and λ_*s*_(*t*) estimated independently and nonparametrically (Unrelated *N*_*e*_(*t*) and λ_*s*_(*t*)) and a conditional model combined with a constant sampling intensity model for sampling times (Constant λ_*s*_(*t*)).

Model	ML	Coef	Q0.025	Median	Q0.975
Unrelated *N*_*e*_(*t*) and λ_*s*_(*t*)	-821	—	—	—	—
Constant λ_*s*_(*t*)	-616	—	—	—	—
{*γ*(*t*)}	-597	*γ*(*t*)	0.28	0.46	0.71
{*γ*(*t*), −*t*}	-601	*γ*(*t*)	0.30	0.49	0.83
		−*t*	-0.58	0.27	1.46
{*γ*(*t*), −*t*, −*t* ⋅ *γ*(*t*)}	—	*γ*(*t*)	1.01	1.76	3.32
		−*t*	-0.88	0.18	1.02
		−*t* ⋅ *γ*(*t*)	0.89	1.65	3.11
{*γ*(*t*), −*t*, −*t*^2^}	-596	*γ*(*t*)	0.47	1.00	1.80
		−*t*	2.02	9.05	20.63
		−*t*^2^	-13.08	-5.58	-1.09

Having concluded that posterior predictive checks are underpowered in our setting (see Section B.2.3 in S1 Text), we use our model selection approach applied to a fixed phylogenetic tree obtained using a fast approximate maximum likelihood method implemented in TreeTime software package [36]. Recall that our INLA implementation of BNPR-PS does not allow for interaction covariates, so we compute marginal likelihoods, shown in Table 4, only for models without interactions. The quadratic model has the highest marginal likelihood, but the marginal likelihood of the preferential sampling model without additional covariates is only slightly lower. Again, marginal likelihoods of all preferential sampling models but with the constant sampling intensity are higher than the likelihood of the unrelated *N*_*e*_(*t*) and λ_*s*_(*t*) model.

As another way to compare adequacy of preferential sampling models, we overlay our reconstructed effective population size trajectories and Ebola weekly incidence time series. We use a sum of confirmed and probable case counts from the supplementary data of [35]. Assuming a susceptible-infectious-removed (SIR) model, an approximate structured coalescent model, and taking into account the fact that the number of susceptible individuals never decreased appreciably during the Ebola outbreak, we can interpret effective population size as a quantity proportional to Ebola incidence [37, 3]. Figure C-1 in S1 Text shows aligned incidence time series and posterior summaries of effective population size for all considered coalescent models. All models produce reasonable agreement between incidence and effective population size during the increase of incidence. However, the end of the outbreak is captured better by preferential sampling models, with the quadratic model {*γ*(*t*), −*t*, −*t*^2^} outperforming the other preferential sampling models.

**Fig 8:**
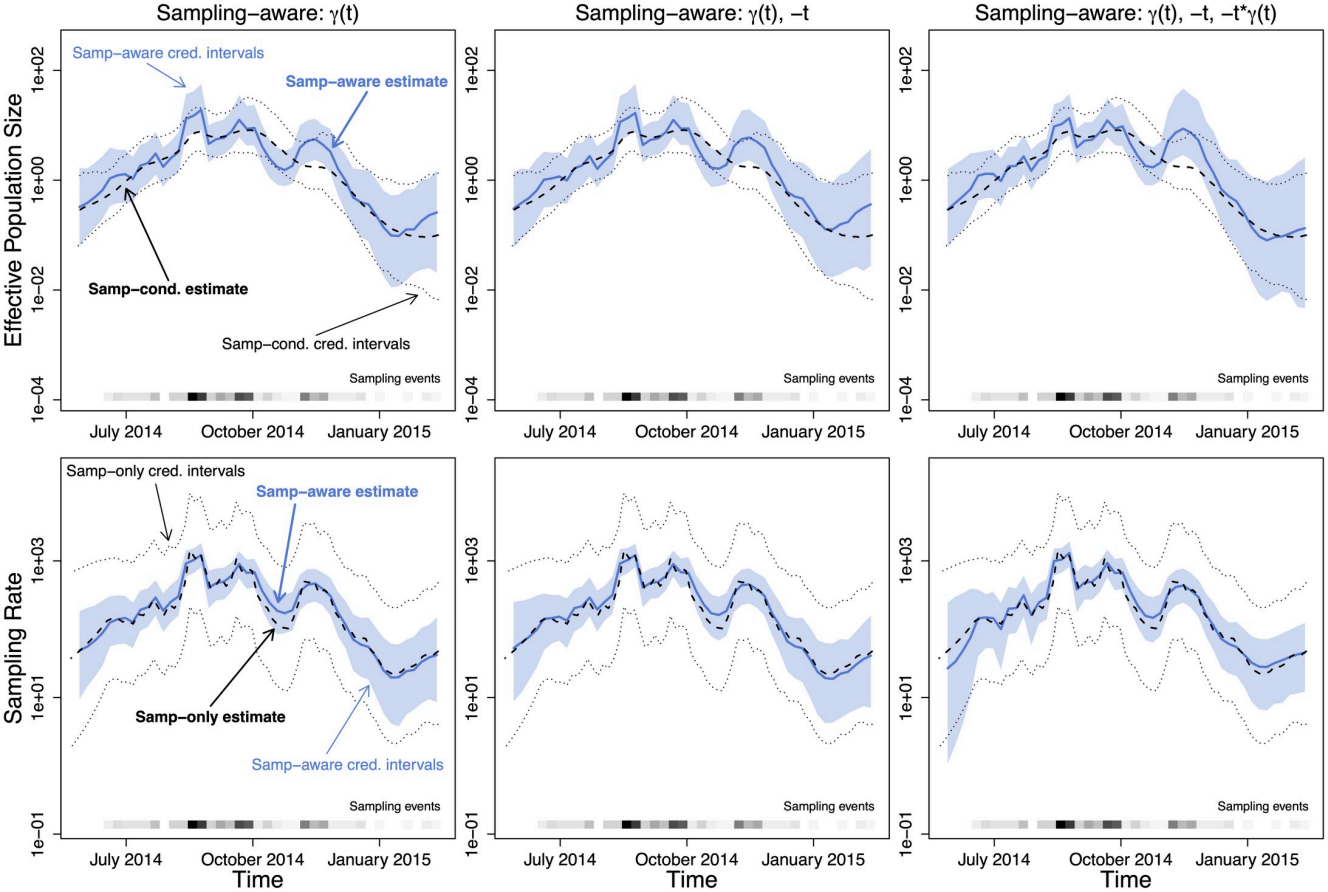
Effective population size and sampling rate reconstructions for the Liberia Ebola dataset. *Upper row*: Dashed lines and dotted black lines are the pointwise posterior effective population size estimates and credible intervals of the sampling-conditional model. The blue lines and the light blue regions are the pointwise posterior effective population size estimates and credible intervals of that column’s sampling-aware model. *Lower row*: Dashed lines and dotted black lines are the pointwise posterior sampling rate estimates and credible intervals of a nonparametric sampling-time-only model. The blue lines and the light blue regions are the pointwise posterior sampling rate estimates and credible intervals of that column’s sampling-aware model.

We apply the same models to the Liberia Ebola dataset, summarized across the upper row of Fig 8 and in Table 5. We note that the {*γ*(*t*)} and {*γ*(*t*), −*t*} models perform very similarly, but the {*γ*(*t*), −*t*} model has slightly wider pointwise credible intervals in places. This is consistent with the coefficients, as the credible interval for the −*t* term contains 0. The {*γ*(*t*), −*t*, −*t* ⋅ *γ*(*t*)} model has even wider pointwise credible intervals, and the credible intervals for the coefficients all contain 0. However, the {*γ*(*t*), −*t*} has the highest marginal likelihood, closely followed by the simple preferential sampling model {*γ*(*t*)}. Again, all marginal likelihoods, reported in Table 5, are computed using a fixed phylogeny estimated using TreeTime. We also note that in the {*γ*(*t*)} model, the median estimate for the coefficient for *γ*(*t*) is close to 1.0, suggesting direct proportional sampling.

**Table 5:** Summary of Liberia Ebola sequence data inference. Posterior distribution quantile summaries for BEAST implementation of BNPR-PS models: with no covariates (model: {*γ*(*t*)}), with an ordinary covariate (model: {*γ*(*t*), −*t*}), with both an ordinary and interaction covariate (model: {*γ*(*t*), −*t*, −*t* ⋅ *γ*(*t*)}). Using a fixed tree, we also include marginal likelihoods (MLs) for all of the above models with no interactions, plus a model with *N*_*e*_(*t*) and λ_*s*_(*t*) estimated independently and nonparametrically (Unrelated *N*_*e*_(*t*) and λ_*s*_(*t*)) and a conditional model combined with a constant sampling intensity model for sampling times (Constant λ_*s*_(*t*)).

Model	ML	Coef	Q0.025	Median	Q0.975
Unrelated *N*_*e*_(*t*) and λ_*s*_(*t*)	-825	—	—	—	—
Constant λ_*s*_(*t*)	-567	—	—	—	—
{*γ*(*t*)}	-527	*γ*(*t*)	0.53	0.78	1.20
{*γ*(*t*), −*t*}	-524	*γ*(*t*)	0.53	0.81	1.23
		−*t*	-3.39	-0.74	1.84
{*γ*(*t*), −*t*, −*t* ⋅ *γ*(*t*)}	—	*γ*(*t*)	-0.07	0.40	1.51
		−*t*	-3.26	-0.31	2.67
		−*t* ⋅ *γ*(*t*)	-2.98	-1.21	1.20

As in the previous section, we compare the sampling rates we derive from our BEAST runs to a nonparametric INLA-based estimate of the sampling rate. Fig 7 (lower row) and Fig 8 (lower row) show the comparisons. The two methods produce very similar estimates, and again the sampling-aware methods have thinner credible intervals due to incorporating additional information from the coalescent likelihood.

All coalescent models produce reasonable agreement between estimated effective population size trajectories and Ebola incidence time series in Libera (see Figure C-2 in S1 Text). The model without preferential sampling looks the best in this comparison, mostly because the incidence curve does not support multiple “ups” and “downs” in effective population size trajectories estimated under the preferential sampling models. We note that although *N*_*e*_(*t*) tracks incidence fairly well in our Ebola analyses, such correspondence requires many assumptions to hold, which is not the case in many applications.

## Discussion

Currently, few phylodynamic methods incorporate sampling time models in order to address model misspecification and take advantage of the additional information contained in sampling times in preferential sampling contexts. Even fewer methods implement sampling time models by appropriately integrating over genealogies relating the sampled genetic sequences and performing inference directly from these sequence data. We extend previous sampling time models to incorporate time-varying covariates in order to allow the sampling model to be more flexible under different scientific circumstances. We implement this sampling time model into the MCMC software BEAST, and also implement an elliptical slice sampler into BEAST for efficient MCMC draws of grid-based effective population size parameterizations.

However, the additional flexibility of the sampling time model comes with additional uncertainty around which set of covariates is the best one for a given scientific context. There is a danger of including too many irrelevant covariates into the model, leading to identifiability problems. If one wishes to pursue a preferential sampling model with a large number of potential covariates, we recommend using Bayesian regularization priors to guard against overfitting [38]. Regardless of the number of covariates, one needs to probe adequacy of the preferential sampling model Although posterior predictive checks [27] lacked sufficient power to discriminate between models in all of our applications, using marginal likelihoods to compare preferential sampling models to the model with unrelated *N*_*e*_(*t*) and λ(*t*) offers a promising alternative. We have implemented this model comparison approach for a fixed tree and plan to port this approach to BEAST in the future.

Another approach to extending and increasing the flexibility of the sampling model is to decouple the fixed temporal relationship between effective population size and sampling intensity. Introducing an estimated lag parameter to the sampling time model would allow for cause-and-effect phenomena and delays to be accounted for within the model. Incorporating an estimated lag parameter would also allow for an additional avenue of model verification. Under most imaginable circumstances, if there is a relationship between the effective population size and sampling frequency, changes to the population size would effect sampling frequency with zero or positive delay. Estimating a credibly negative lag would be a possible indicator that some element of the model or data is worth re-examining.

In terms of flexibility, the ideal sampling time model would be a separate Gaussian latent field distinct from the (log) effective population size. However, methods for primarily phylodynamic inference with this feature would suffer from severe identifiability problems. One approach that would retain most of the flexibility of the separate Gaussian field while also retaining the identifiability of the original model would be to model the (log) effective population size and sampling intensities as *correlated* Gaussian processes. Estimating the correlation parameter between the two processes would allow for estimation of the preferential sampling strength. Finally, since preferential sampling can occur both in space and in time [39], it would be natural to extend our framework to structured/not well mixed populations [40].

## Supporting information

S1 Text The file S1_Text.pdf contains appendices with additional simulation and real data analysis details. (PDF)
