# Supplementary material for: Estimating effective population size changes from preferentially sampled genetic sequences
Source: PLoS Comput Biol. 2020 Oct 12;16(10):e1007774. doi: 10.1371/journal.pcbi.1007774 (PMC7580988; doi:10.1371/journal.pcbi.1007774)
Supplement: S1 Text — (PDF) [file pcbi.1007774.s001.pdf]

Supplementary Materials

for "Estimating effective population size changes from preferentially sampled genetic sequences" by

Michael D. Karcher, Luiz Max Carvalho, Marc A. Suchard, Gytis Dudas and Vladimir N. Minin

A Appendix: Additional Sequence Data Results

A.1 Seasonal Influenza

We consider one additional model for the USA/Canada influenza data with log-intensity,

$$\beta_0 + \beta_1\gamma(t) + \beta_2 I_{\text{winter}}(t) + \beta_3 I_{\text{autumn}}(t) + \beta_4 I_{\text{summer}}(t) \\ + \delta_2 I_{\text{winter}}(t)\cdot\gamma(t) + \delta_3 I_{\text{autumn}}(t)\cdot\gamma(t) + \delta_4 I_{\text{summer}}(t)\cdot\gamma(t),$$

abbreviated $\{\gamma(t),\, I_{\text{winter}},\, I_{\text{autumn}},\, I_{\text{summer}},\, I_{\text{winter}}\cdot\gamma(t),\, I_{\text{autumn}}\cdot\gamma(t),\, I_{\text{summer}}\cdot\gamma(t)\}$, or more succinctly as $\{\gamma(t),\, I_{\text{winter}},\, I_{\text{autumn}},\, I_{\text{summer}},\, \text{interactions}\}$. The results are summarized in Figure A-1 and Table A-1. We see that only the coefficients for $\gamma(t)$, I_{winter}, and I_{autumn} have credible intervals that do not contain zero, suggesting that additional terms are not necessary.

A.2 Ebola Outbreak

We consider three additional models for our subsample of 200 sequences from the Sierra Leone Ebola outbreak data with log-intensities,

$$\beta_0 + \beta_1\gamma(t) + \beta_2\cdot(-t) + \beta_3\cdot(-t^2) \\ + \delta_2\gamma(t)\cdot(-t) + \delta_3\gamma(t)\cdot(-t^2), \text{ and} \\ \beta_0 + \beta_1\gamma(t) + \delta_2\gamma(t)\cdot(-t) + \delta_3\gamma(t)\cdot(-t^2),$$

Model	Coef	Q0.025	Median	Q0.975
$\{\gamma(t), I_{\text{winter}}, I_{\text{autumn}}, I_{\text{summer}}, \text{interactions}\}$	$\gamma(t)$	0.64	1.20	1.97
	I_{winter}	1.83	3.48	5.58
	I_{autumn}	1.31	3.16	5.28
	I_{summer}	-0.15	2.08	4.52
	$I_{\text{winter}}\cdot\gamma(t)$	-1.08	-0.29	0.34
	$I_{\text{autumn}}\cdot\gamma(t)$	-1.00	-0.14	0.53
	$I_{\text{summer}}\cdot\gamma(t)$	-1.24	-0.24	0.92

Table A-1: **Summary of USA/Canada influenza data inference.** Posterior distribution quantile summaries for SampESS with seasonal indicator and interaction covariates (model: $\{\gamma(t), I_{\text{winter}}, I_{\text{autumn}}, I_{\text{summer}},\text{interactions}\}$).

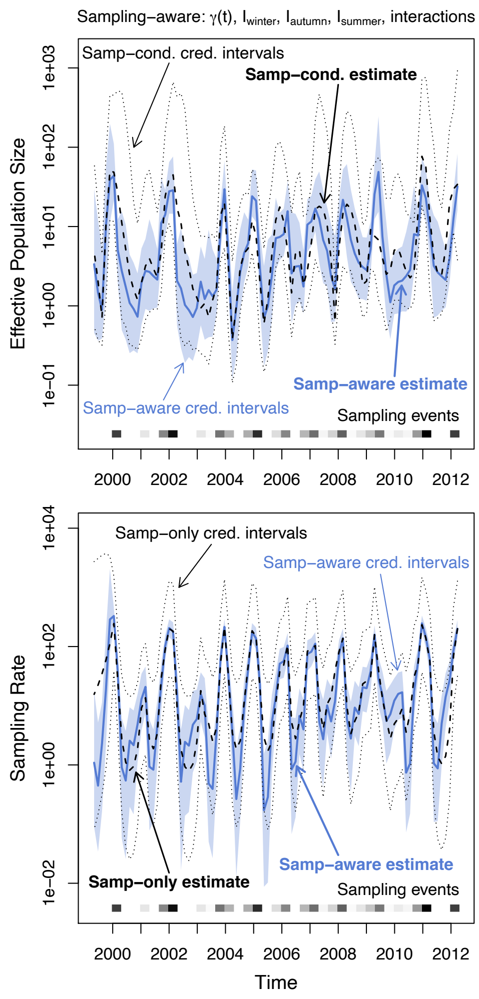

Figure A-1: **Effective population size and sampling rate reconstructions for the USA and Canada influenza dataset.** *Upper row*: Dashed lines and dotted black lines are the pointwise posterior effective population size estimates and credible intervals of the sampling-conditional model. The blue line and the light blue region are the pointwise posterior effective population size estimates and credible intervals of that column's sampling-aware model. *Lower row*: Dashed lines and dotted black lines are the pointwise posterior sampling rate estimates and credible intervals of a nonparametric sampling-time-only model. The blue line and the light blue region are the pointwise posterior sampling rate estimates and credible intervals of that column's sampling-aware model.

abbreviated as $\{\gamma(t), -t, -t^2, -t\cdot\gamma(t), -t^2\cdot\gamma(t)\}$, and $\{\gamma(t), -t\cdot\gamma(t), -t^2\cdot\gamma(t)\}$, respectively. The results are summarized in Figure A-2 and Table A-2. We see that the coefficients for $\gamma(t)$, $-t$, and $-t^2$ tend to have credible intervals that do not contain zero (except for the interaction-only model $\{\gamma(t), -t\cdot\gamma(t), -t^2\cdot\gamma(t)\}$), but the other terms do not, suggesting that the additional terms are not necessary.

**Model**	**Coef**	**Q0.025**	**Median**	**Q0.975**
$\{\gamma(t), -t, -t^2, -t\cdot\gamma(t), -t^2\cdot\gamma(t)\}$	$\gamma(t)$	0.71	2.20	4.69
	$-t$	1.21	9.75	20.29
	$-t^2$	-12.67	-6.00	-0.79
	$-t\cdot\gamma(t)$	-2.72	0.95	6.49
	$-t^2\cdot\gamma(t)$	-2.64	0.72	3.26
$\{\gamma(t), -t\cdot\gamma(t), -t^2\cdot\gamma(t)\}$	$\gamma(t)$	-3.00	-1.39	2.08
	$-t\cdot\gamma(t)$	-11.30	-6.59	2.00
	$-t^2\cdot\gamma(t)$	-0.16	4.90	8.16

Table A-2: **Summary of Sierra Leone Ebola sequence data inference.** Posterior distribution quantile summaries for SampESS with models: $\{\gamma(t), -t, -t^2, -t\cdot\gamma(t), -t^2\cdot\gamma(t)\}$, and $\{\gamma(t), -t\cdot\gamma(t), -t^2\cdot\gamma(t)\}$.

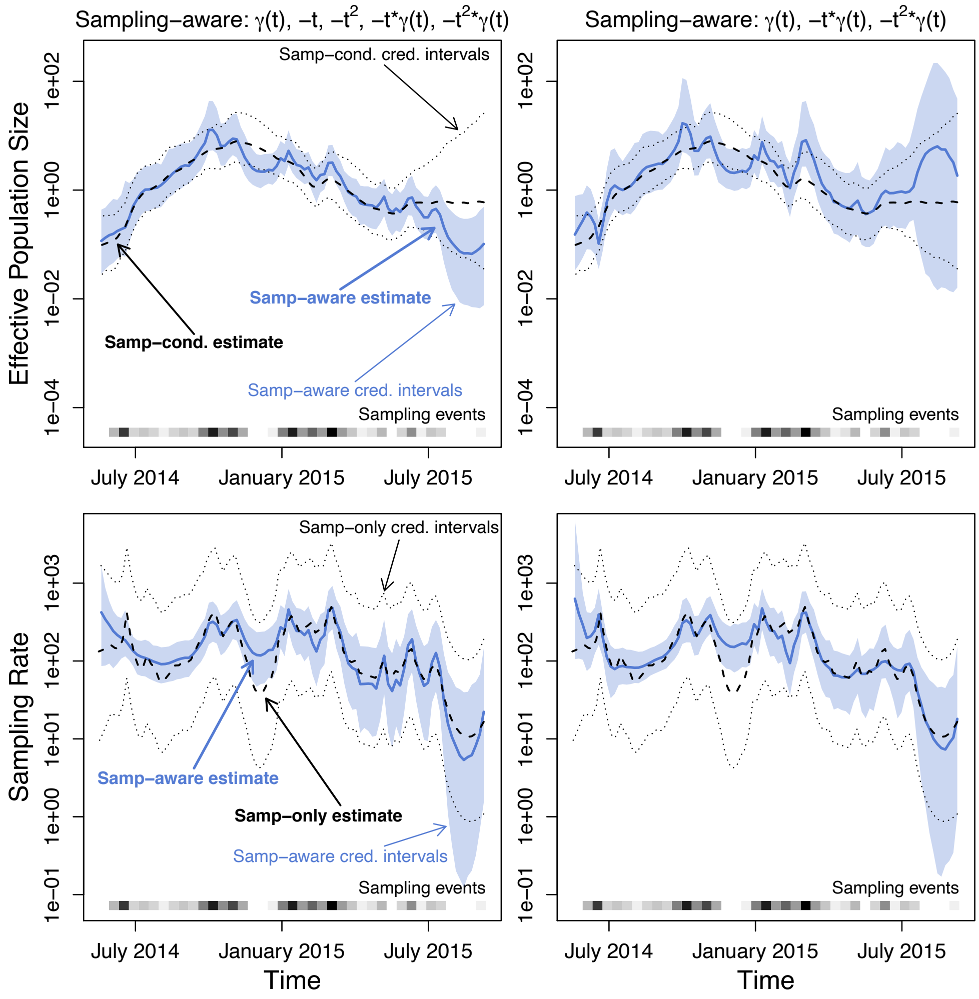

Figure A-2: **Effective population size and sampling rate reconstructions for the Sierra Leone Ebola dataset.** *Upper row*: Dashed lines and dotted black lines are the pointwise posterior effective population size estimates and credible intervals of the sampling-conditional model. The blue line and the light blue region are the pointwise posterior effective population size estimates and credible intervals of that column's sampling-aware model. *Lower row*: Dashed lines and dotted black lines are the pointwise posterior sampling rate estimates and credible intervals of a nonparametric sampling-time-only model. The blue line and the light blue region are the pointwise posterior sampling rate estimates and credible intervals of that column's sampling-aware model.

B Appendix: Validation, Model Checks, and Model Selection

B.1 Methods

B.1.1 Transformed Exponentials

Suppose random variable $X \sim \text{Exp}(1)$, and thus its PDF is $f_X(x) = \exp(-x)$. Define $g_\lambda(u) = \int_0^u \lambda(t)dt$ for nonnegative $\lambda(\cdot)$ integrable on $[0, \infty)$. Then $g_\lambda(u)$ is monotonic non-decreasing, so $g_\lambda^{-1}(\cdot)$ is well-defined almost everywhere. If we let $U = g_\lambda^{-1}(X)$, then the PDF of U is $f_U(u) = \lambda(u)\exp(-\int_0^u \lambda(t)dt)$.

We then have two useful results. If we wish to sample U, we may do so by sampling an Exp(1) random variable X, then apply the transformations $U = g_\lambda^{-1}(X)$, which will result in the desired distribution. There generally is not an explicit, closed-form solution for $g^{-1}(\cdot)$, but it can be implicitly solved using root-finding methods and, if necessary, numerical integration. Conversely, if we wish to recover the original Exp(1) random variable X from U, we can apply the transformations $X = g_\lambda(U)$.

B.1.2 Heterochronous Coalescent Time Transformation

Consider the heterochronous coalescent model, as presented in Section 2 of the main text. Griffiths and Tavaré [1994a] show that for isochronous data, the sequence of coalescent events of a genealogy (and allowing variable effective population size) is a continuous time Markov chain and that the function $A_n(t)$, representing the number of distinct ancestors at time t and called the *ancestral process*, is a pure death process starting at value n at time 0 and decreasing by one at every coalescent event proceeding into the past.

We seek to extend this framework to allow heterochronous genealogies as well. Consider a Wright-Fisher population with population $N(i)$, i generations in the past. We assume that sampled individuals cannot be ancestors to future sampled individuals, so if we sample an individual at generation i, we segregate that individual from the other $N(i)$ individuals in the population until the sampled individual "selects" an ancestor in generation $i+1$, at which point the usual Wright-Fisher process proceeds until another individual is sampled farther in the past. Suppose we have a fixed schedule of n individuals sampled at generations $g_1 \leq g_2 \leq \ldots \leq g_n$, and we consider any particular generation i, having counted k coalescent events between generation 0 and generation i. Let $b_i = \sum_{i=1}^n 1_{[g_i > i]}$ represent the number of individuals that are sampled farther into the past than generation i. In an isochronous scenario, b_i would be 0 for all i, and the number of distinct lineages at generation i would be $n-k$. However, here we suppose that $b_i > 0$. We see that if there are no individuals sampled at generations i or $i+1$, then this iteration of the Wright-Fisher process is identical to an iteration of an isochronous Wright-Fisher process with the same population and $n-k-b_i$ distinct lineages. If there is an individual sampled at generation $i+1$, the outcome is the same since we can safely ignore the (segregated) sampled individual until iterating from generation $i+1$ to $i+2$. If there is an individual sampled at generation i, then we consider

the (segregated) sampled individual to be an additional distinct lineage, but we see the iteration still behaves as if it were an iteration of an isochronous Wright-Fisher process with $n - k - b_i$ distinct lineages.

We now switch to continuous time, applying our heterochronous distinct lineage counts into the results from [Griffiths and Tavaré, 1994a]. Let $b(t) = \sum_{i=1}^{n} 1_{[s_i > t]}$ be the count of samples that occur farther into the past than time t. Let $B_n(t) = n - k(t)$, where $k(t)$ is the number of coalescent events between time 0 and time t. Under isochronous sampling, $B_n(t) = A_n(t)$ is the ancestral process. Under heterochronous sampling, $B_n(t)$ is merely the pure death process that is directly analogous to $A_n(t)$. Substituting our results from the heterochronous Wright-Fisher process into the key results reveals the transition rates for $B_n(t)$,

$$\Pr(B_n(t+h) = j \mid B_n(t) = i) = \begin{cases} \binom{i-b(t)}{2} \frac{1}{N_e(t)} h + o(h), & j = i - 1 \\ 1 - \binom{i-b(t)}{2} \frac{1}{N_e(t)} h + o(h), & j = i \\ 0 & \text{otherwise,} \end{cases}$$

and the joint density for the Markov chain of coalescent events,

$$\Pr(\mathbf{g} \mid N_e(t), \mathbf{s}) = \prod_{k=2}^{n} \left[\lambda_k(t_{k-1}) \exp\left(- \int_{t_k}^{t_{k-1}} \lambda_k(t) dt \right) \right],$$

where $\lambda_k(t) = \binom{k-b(t)}{2} \frac{1}{N_e(t)}$.

Following the results from [Griffiths and Tavaré, 1994a], we note that the terms in the product are in the form of transformed exponentials, and can be sampled by transforming $n-1$ independent, identically distributed (i.i.d.) Exp(1) random variables. Finally, we note that we can recover these exact $n-1$ i.i.d. Exp(1) random variables by applying the inverse transformation.

B.1.3 Coalescent Posterior Predictive Checks

We consider the Bayesian approach for phylodynamic analysis laid out in Section 2 of the main text. Similar to Gelman et al. [1996]'s mixed predictive distribution approach, we simulate data and certain latent variables from our models, informed by our posterior sample, in order to judge how well those models adhere to observed and inferred realities. In the context of our posterior with no sampling time model, we replicate $\{\mathbf{y}_i^{\text{rep}}\}_{i=1}^{N}$ and $\{\mathbf{g}_i^{\text{rep}}\}_{i=1}^{N}$ according to this joint posterior,

$$\Pr(\mathbf{y}^{\text{rep}}, \mathbf{g}^{\text{rep}}, \boldsymbol{\gamma}, \kappa, \boldsymbol{\theta} \mid \mathbf{y}, \mathbf{s}) \propto \Pr(\mathbf{y}^{\text{rep}} \mid \mathbf{g}^{\text{rep}}, \boldsymbol{\theta}) \Pr(\mathbf{g}^{\text{rep}} \mid \boldsymbol{\gamma}, \mathbf{s}) \Pr(\boldsymbol{\gamma}, \kappa, \boldsymbol{\theta} \mid \mathbf{y}, \mathbf{s}), \quad (6)$$

simulating from the coalescent $\Pr(\mathbf{g}^{\text{rep}} \mid \boldsymbol{\gamma}, \mathbf{s})$ and (if necessary, see below) the substitution model $\Pr(\mathbf{y}^{\text{rep}} \mid \mathbf{g}^{\text{rep}})$. We sample the final term on the right side via MCMC.

With posterior-sampled replicates available, we construct a discrepancy D_c [Gelman et al.,

1996, Sinharay and Stern, 2003] on the observables and the inferred latent variables. Let $G(\mathbf{g}, \boldsymbol{\gamma})$ be the transformation (explored in the previous section) that, given the correct effective population trajectory, and valid assumptions for the coalescent model, will produce a sample of $n-1$ i.i.d. Exp(1)-distributed random variables. Let K be the Kolmogorov-Smirnov statistic [Massey Jr, 1951],

$$K_{\mathrm{Exp}(1)}(\mathbf{e}) = \sup_{x \in \mathbb{R}} |F_{\mathbf{e}}(x) - F_{\mathrm{Exp}(1)}(x)|, \tag{7}$$

where $F_{\mathbf{e}}(x)$ is the empirical cumulative distribution function (ECDF) of $\mathbf{e}$, and $F_{\mathrm{Exp}(1)}(x)$ is the true cumulative distribution function (CDF) of the Exp(1) distribution. We define

$$D_c(\mathbf{y}, \mathbf{g}, \mathbf{s}, \boldsymbol{\gamma}, \kappa) = K_{\mathrm{Exp}(1)}(G(\mathbf{g}, \boldsymbol{\gamma})).$$

Then when we run MCMC, we then compare the *observed discrepancies*,

$$\{D_c(\mathbf{y}, \mathbf{g}_i, \mathbf{s}, \boldsymbol{\gamma}_i, \kappa_i)\}_{i=1}^N,$$

to the *replicate discrepancies*,

$$\{D_c(\mathbf{y}_i^{\mathrm{rep}}, \mathbf{g}_i^{\mathrm{rep}}, \mathbf{s}, \boldsymbol{\gamma}_i, \kappa_i)\}_{i=1}^N.$$

Note that the D_c we constructed does not depend on $\mathbf{y}^{\mathrm{rep}}$, so we can save computation time by not simulating $\mathbf{y}^{\mathrm{rep}} \mid \mathbf{g}^{\mathrm{rep}}$. If we wish to check the sampling-aware posterior with the sampling time model, the replicate posterior remains mostly the same as in Equation 6, but the final term becomes $\Pr(\boldsymbol{\gamma}, \kappa, \boldsymbol{\beta}, \boldsymbol{\theta} \mid \mathbf{y}, \mathbf{s}, \mathcal{F})$ to match the sampling-aware posterior.

One method we have to compare the observed and replicate discrepancies is the posterior predictive p-value [Gelman et al., 1996]. We calculate the posterior predictive p-value by finding the proportion of MCMC iterations where the replicated discrepancy values are larger than its corresponding observed discrepancy value. The smaller the posterior predictive p-value, the more unusual the observed data is in the context of the chosen model. Note that this posterior predictive p-value does not have the usual frequentist p-value properties such as uniformity under a null model. However, values close to 50% suggest that the current model is adequate, and for discrepancies that become larger as the observed data becomes less likely given a set of parameters, the posterior predictive p-value tends to be smaller, to some degree, under under inadequate models [Gelman et al., 1996].

B.1.4 Sampling Posterior Predictive Checks

Similarly to the previous section, we replicate $\{\mathbf{y}_i^{\mathrm{rep}}\}_{i=1}^N$, $\{\mathbf{g}_i^{\mathrm{rep}}\}_{i=1}^N$, and $\{\mathbf{s}_i^{\mathrm{rep}}\}_{i=1}^N$ according to this joint posterior,

$$\begin{aligned}\Pr(\mathbf{y}^{\mathrm{rep}}, \mathbf{g}^{\mathrm{rep}}, \mathbf{s}^{\mathrm{rep}}, \boldsymbol{\gamma}, \kappa, \boldsymbol{\beta}, \boldsymbol{\theta} \mid \mathbf{y}, \mathbf{s}) \propto \Pr(\mathbf{y}^{\mathrm{rep}} \mid \mathbf{g}^{\mathrm{rep}}) \Pr(\mathbf{g}^{\mathrm{rep}} \mid \boldsymbol{\gamma}, \mathbf{s}^{\mathrm{rep}}) \Pr(\mathbf{s}^{\mathrm{rep}} \mid \boldsymbol{\gamma}, \boldsymbol{\beta}) \\ \times \Pr(\boldsymbol{\gamma}, \kappa, \boldsymbol{\beta}, \boldsymbol{\theta} \mid \mathbf{y}, \mathbf{s}),\end{aligned} \tag{8}$$

with $\Pr(\boldsymbol{\gamma}, \kappa, \boldsymbol{\beta}, \boldsymbol{\theta} \mid \mathbf{y}, \mathbf{s})$ sampled via MCMC. We simulate from the sampling model $\Pr(\mathbf{s}^{\text{rep}} \mid \boldsymbol{\gamma}, \beta)$, and, if necessary, the coalescent $\Pr(\mathbf{g}^{\text{rep}} \mid \boldsymbol{\gamma}, \mathbf{s})$, and the substitution model $\Pr(\mathbf{y}^{\text{rep}} \mid \mathbf{g}^{\text{rep}})$.

Suppose we divide the sampling interval into a grid $K_1, \ldots, K_l$, potentially the same grid as used by grid-based priors for the effective population trajectory. The sampling model is inhomogeneous Poisson, so we can bin the numbers of sampling times within each interval $m_1, \ldots, m_l$, each with expected values $E_i = \int_{K_i} \lambda_s(t)dt$. A common approach to problems with independent Poisson bins is a Chi-squared test with statistic $\chi_s^2 = \sum_{i=1}^{l} \frac{(m_i - E_i)^2}{E_i}$ [Pearson, 1900]. We can then define a discrepancy

$$D_{\chi^2}(\mathbf{y}, \mathbf{g}, \mathbf{s}, \boldsymbol{\gamma}, \kappa) = \sum_{i=1}^{l} \frac{(m_i - E_i)^2}{E_i}, \tag{9}$$

for m_i and E_i derived from $\mathbf{s}$ as above.

B.1.5 Marginal likelihoods and Bayes factors

Another tool to aid model comparison are marginal likelihoods, which capture the evidence brought by the data in favor of a given model once one integrates (averages) over the parameters. Let $\mathcal{M}_0$ and $\mathcal{M}_1$ be two models under consideration, with $\boldsymbol{\xi}$ the parameters of interest and $\boldsymbol{y}$ be the observed data. A key quantity, that summarizes support for model i is the marginal likelihood:

$$p(\boldsymbol{y} \mid \mathcal{M}_i) = \int_{\mathcal{X}} L(\boldsymbol{\xi} \mid \boldsymbol{y}, \mathcal{M}_i)\pi(\boldsymbol{\xi} \mid \mathcal{M}_i)\, d\boldsymbol{\xi}. \tag{10}$$

The marginal likelihoods of competing models can then be compared *via* Bayes factors. The Bayes factor between models 0 and 1 is

$$\text{BF}_{01} = \frac{p(\boldsymbol{y} \mid \mathcal{M}_0)}{p(\boldsymbol{y} \mid \mathcal{M}_1)} \tag{11}$$

and quantifies the support in favor of model 0 in comparison to model 1. See Kass and Raftery [1995] for guidance on the interpretation of Bayes factors.

For models fitted using integrated nested Laplace approximation (INLA), we employ the approximation

$$p(\boldsymbol{y} \mid \mathcal{M}) \approx \int_{\boldsymbol{\Theta}} \left. \frac{p(\boldsymbol{y}, \boldsymbol{\xi}, \boldsymbol{\eta})}{\pi_G(\eta \mid \boldsymbol{y}, \boldsymbol{\xi}, \mathcal{M})} \right|_{\boldsymbol{\eta}=\boldsymbol{\eta}^*(\boldsymbol{\xi}|\mathcal{M})} d\boldsymbol{\xi}, \tag{12}$$

where $\boldsymbol{\eta}$ is a vector of latent variables – in our case, $\boldsymbol{\eta} = \gamma(t) = \log N_e(t)$ – and $\boldsymbol{\eta}^*(\boldsymbol{\xi} \mid \mathcal{M})$ is some chose value, typically the posterior mode, and π_G is a Gaussian approximation of the (marginal) posterior $\pi(\boldsymbol{\eta} \mid \boldsymbol{y}, \boldsymbol{\theta}, \mathcal{M})$. For a detailed study of the performance of these approximations, see Hubin and Storvik [2016].

B.2 Results

B.2.1 Fixed-genealogy Simulation Study

In order to evaluate the use of INLA-based Bayesian estimation and marginal likelihood-based for selecting the best model for population size reconstruction, we simulate data under four scenarios:

- Scenario 1: **Uniform**. Sampling is uniform through time, i.e., $\lambda_s(t) = \exp(\beta_0)$;
- Scenario 2: **Preferential**. Sampling proportional to $\gamma(t) = \log N_e(t)$, i.e., $\lambda_s(t) = \exp(\beta_0 + \beta_1\gamma(t))$;
- Scenario 3: **Covariate**. Sampling depends on $\gamma(t)$ and a time-covariate $x(t)$, $\lambda_s(t) = \exp(\beta_0 + \beta_1\gamma(t) + \beta_2 x(t))$;
- Scenario 4: **Unrelated**. Sampling intensity depends on process that is independent of $\gamma(t)$ and $x(t)$, $\lambda_s(t) = g(t)$.

Here, we pick $x(t) = \exp(-0.05t)$ and $g(t) = P(t)$. We fit a suite of four models to each scenario:

- Model 1: conditional model (BNPR) plus uniform sampling; assuming $\lambda_s(t) = \exp(\beta_0)$;
- Model 2: preferential sampling model (BNPR-PS), assuming $\lambda_s(t) = \exp(\beta_0 + \beta_1\gamma(t))$;
- Model 3: preferential sampling with time covariate (BNPR-PS + Cov), assuming $\lambda_s(t) = \exp(\beta_0 + \beta_1\gamma(t) + \beta_2 x(t))$;
- Model 4: conditional model plus a non-parametric model of the sampling rate, assuming $\lambda_s(t) = \exp(\beta_0 + z(t))$, where $z(t)$ is a first-order random walk.

This simulation study is aimed at assessing, simultaneously, the accuracy of population reconstructions under correctly-specified and misspecified scenarios, as well as the ability of INLA-based marginal likelihoods to correctly identify the correct model, assuming it is included in the set of models being considered. We begin by showing the mean absolute error in population reconstructions, under the four scenarios considered, in Figure B-1. Overall, the models behave as expected, with lower MADs being attained by the correctly-specified model. An important feature is the sharp drop in performance experienced by the preferential models (models 2 and 3) when the sampling process depends on a process unrelated to $N_e(t)$ (Scenario 4), shown in the bottom right panel.

Next, we show the mean width of credible intervals (MCIW) in Figure B-2 and we again see that the correct model attains more confident estimates. Moreover, in addition to being wrong in Scenario 4, models 2 and 3 also lead to smaller credibility intervals, meaning these models give wrong estimates with high confidence.

Regarding the "power" of the model selection framework proposed here, Figure B-3 shows that for larger sample sizes, one recovers the correct model with high probability. Note also

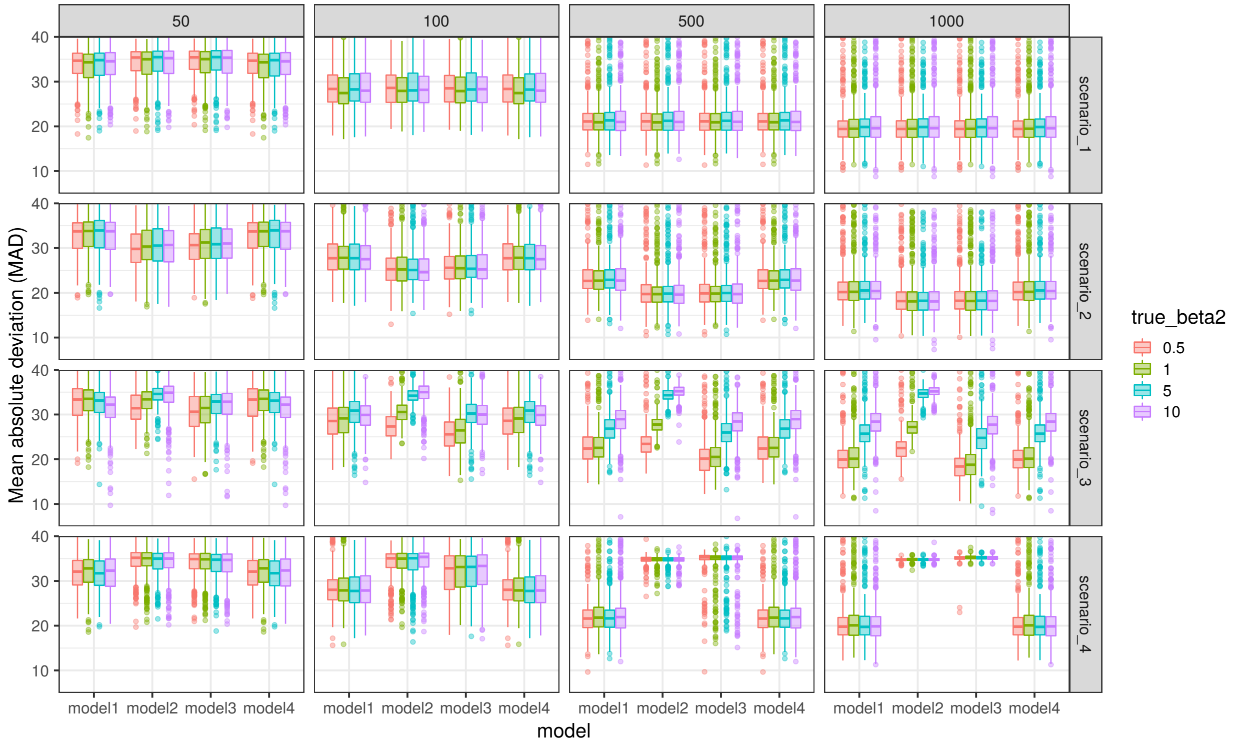

Figure B-1: **Mean absolute deviations (MAD) for the reconstructed $\gamma(t)$.** We show MAD for $\gamma(t)$ for several sample sizes and each of the four models in each of the four data-generating scenarios. Colours show the true value of β_2, when applicable.

that models 3 and 4 are harder to discern for most sample sizes, specially model 4. This is to be expected, as model 4 relies on a non-parametric estimator of the sampling intensity, which likely necessitates more data to achieve informative inferences.

Figure B-4 shows the results of estimating β_2 in Scenario 3 (where model 3 is the correct one) for various sample sizes, in the form of scaled absolute deviations from the true value. Results are what one would expect in that the estimation error decreases with sample size. Coverage of the true values by 95% credibility intervals is also close to the nominal value for most combinations of true β_2 and sample size (Figure B-5).

B.2.2 Simulations to evaluate posterior predictive checks

Genealogy Inference We perform a simulation study in order to explore the capabilities of the posterior predictive checks proposed above in Sections B.1.3 and B.1.4. We begin with a simplified version of the phylodynamic data-to-inference methodology. Here we take genealogies to be our observed data (and move on to inference based on observed sequence data in the next section). We simulate sampling times according to inhomogeneous Poisson processes with different intensity trajectories via a time-transformation method [Çinlar, 1975] as we implemented in our R package `phylodyn` [Karcher et al., 2017]. Give sampling time

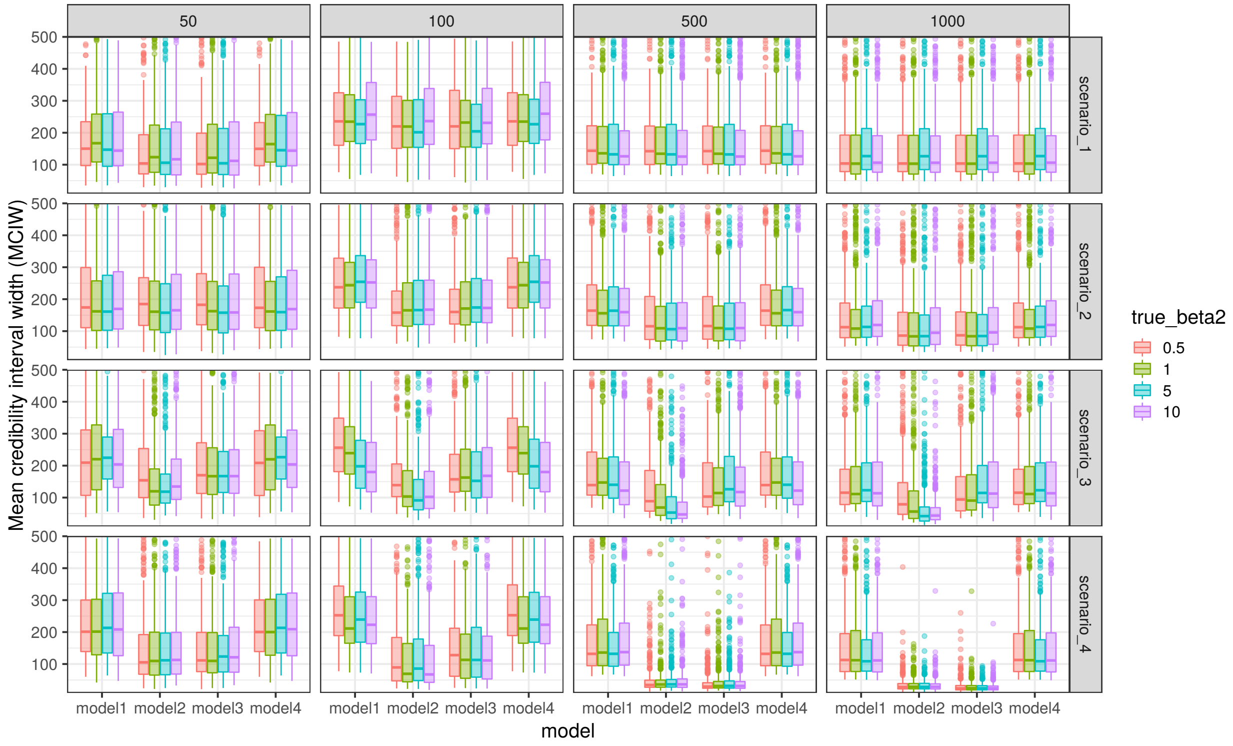

Figure B-2: **Mean credibility interval width (MCIW) for the reconstructed $\gamma(t)$.** We show MCIW (95% BCI) for $\gamma(t)$ for several sample sizes and each of the four models in each of the four data-generating scenarios. Colours show the true value of β_2, when applicable.

data, we simulate from the coalescent model using a similar time-tranformation method for the coalescent [Slatkin and Hudson, 1991], again as implemented in `phylodyn`. For all of our fixed-tree simulations, we use an effective population size trajectory designed to mimic the seasonal effective population size changes of a seasonal disease such as influenza in North America [Zinder et al., 2014], defined as follows:

$$N_{e,l,u,p,o}(t) = \begin{cases} l + \frac{(u-l)}{1+\exp\{2[3-(\frac{t+o}{p} \pmod{12}))]\}}, & \text{if } \frac{t+o}{p} \pmod{12} \leq 6, \\ l + \frac{(u-l)}{1+\exp\{2[3+(\frac{t+o}{p} \pmod{12})-12]\}}, & \text{if } \frac{t+o}{p} \pmod{12} > 6. \end{cases} \tag{13}$$

Specifically, we use $N_{e,10,100,12,0}(t)$ which is most comparable to an influenza effective population size trajectory as measured in units of weeks, with $t = 0$ representing the summer effective population size minimum. We compare the results of our posterior predictive checks across different sampling scenario and choice-of-posterior combinations.

In our first scenario, we simulate 500 sampling times, distributed according to a uniform distribution between $t = 0$ and $t = 24$ (weeks), and simulate a genealogy with effective population size $N_{e,10,100,12,0}(t)$. We infer the underlying effective population size trajectory

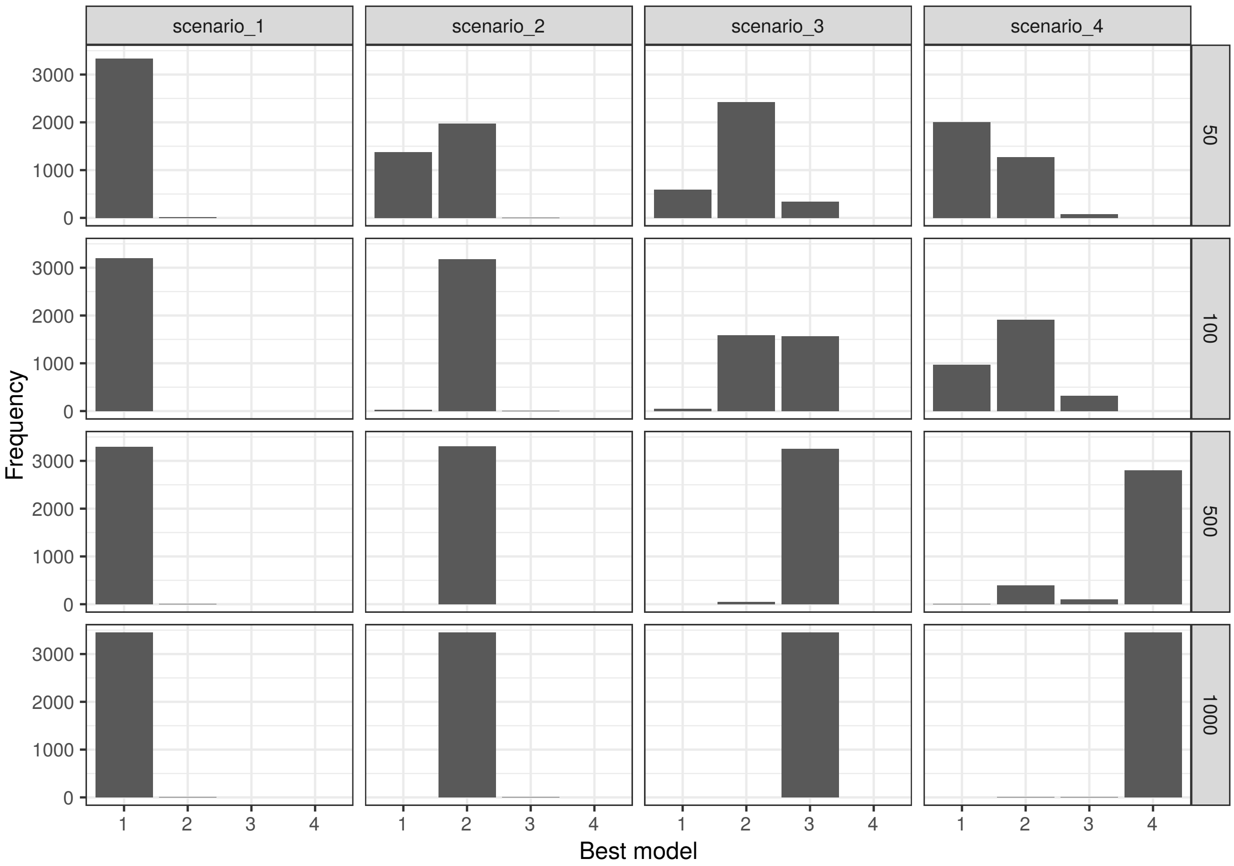

Figure B-3: **Model selection *via* marginal likelihoods.** Bars show the frequency with which each model was the best fitting model (highest marginal likelihood) for each scenario and sample size.

with a sampling-conditional posterior using a Markov chain Monte Carlo (MCMC) method with an elliptical slice sampling transition kernel (ESS) [Murray et al., 2010] as implemented in `phylodyn` (illustrated in the first row, first column of Figure B-6) We use the MCMC output to generate replicate coalescent data as laid out in Section B.1.3 and calculate our coalescent discrepancy D_c for the observed MCMC results as well as for the replicated results. We plot the discrepancy comparison in the second row, first column of Figure B-6, and note that the posterior predictive p-value is 0.58, which is close to 0.5, correctly suggesting that the model is adequate.

We proceed with several additional scenarios. We simulate 514 sampling times between $t = 0$ and $t = 24$ (weeks), distributed proportionally to the effective population size, with sampling log-intensity $\log[\lambda_c(t)] = -0.97 + N_{e,10,100,12,0}(t)$. We infer the underlying effective population size trajectory with a sampling-aware posterior (illustrated in the second column of Figure B-6), with sampling time model $\log[\lambda_s(t)] = \beta_0 + \beta_1 \cdot \boldsymbol{\gamma}(t)$. We calculate the posterior predictive p-value as 0.59, again correctly suggesting adequacy. We also simulate

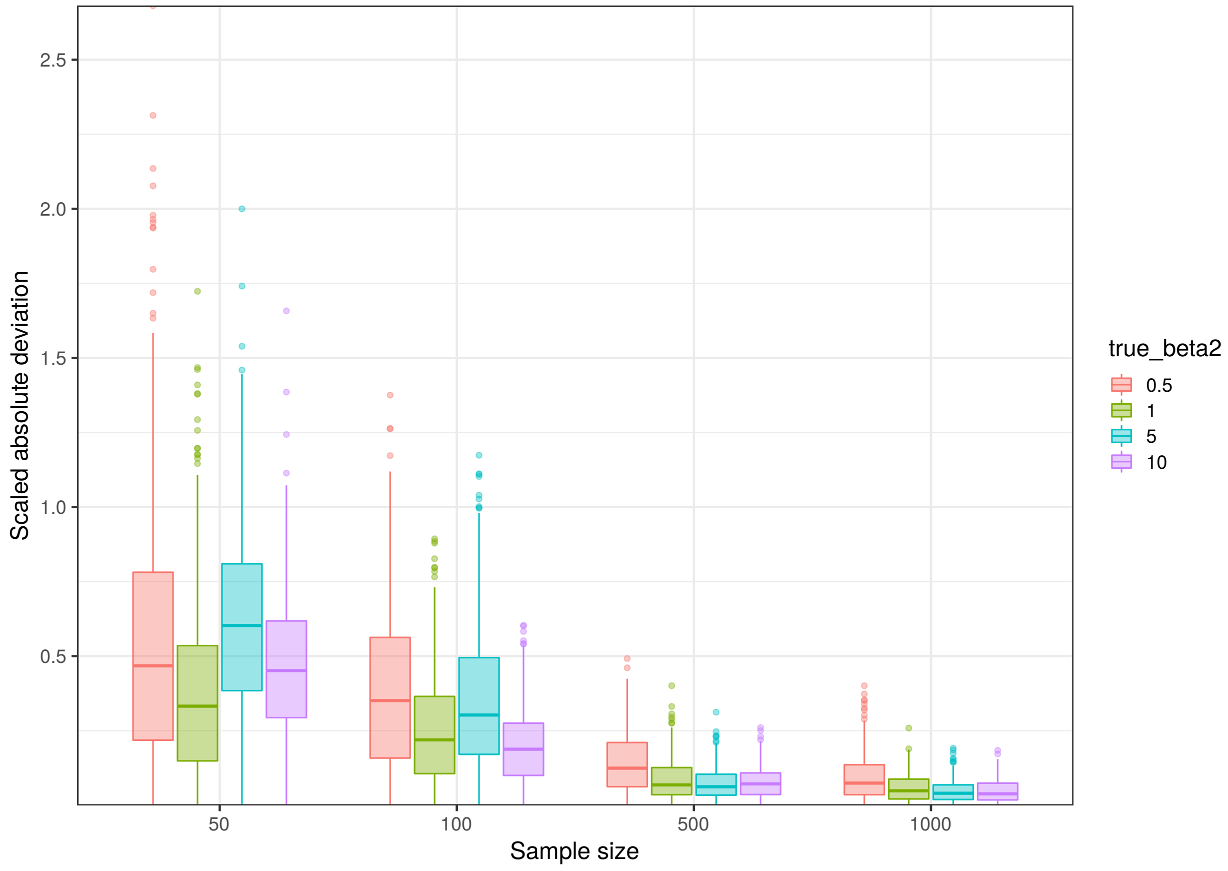

Figure B-4: **Scaled absolute deviation in the estimation of β_2.** We show boxplots of the absolute deviation in the estimate of β_2 scaled by the true value in Scenario 2 for several sample sizes. Colours show the true value of β_2.

509 sampling times between $t = 0$ and $t = 48$ (weeks), distributed proportionally to a piecewise constant function $P(t)$ (illustrated in the second column of Figure B-7) unrelated to the effective population size, with log-sampling intensity $\log[\lambda_c(t)] = -1.67 + P(t)$. We infer the underlying effective population size trajectory using two different methods. We use the sampling-conditional method (illustrated in the third column of Figure B-6) and the sampling-aware method (illustrated in the fourth column of Figure B-6) with sampling log-intensity $\log[\lambda_s(t)] = \beta_0 + \beta_1 \cdot \boldsymbol{\gamma}(t)$. The sampling-conditional posterior predictive p-value becomes 0.46, suggesting that this method (which only considers the coalescent model) does produce an adequate estimate of the effective population size trajectory. The sampling-aware posterior predictive p-value becomes zero, suggesting that this method produced a very poor estimate of the effective population size trajectory (very visible in Figure B-6). This is likely due to the sampling time model mistaking fluctuations in sampling intensity for information about the effective population size trajectory, illustrating the importance of model checking when the true sampling model is uncertain.

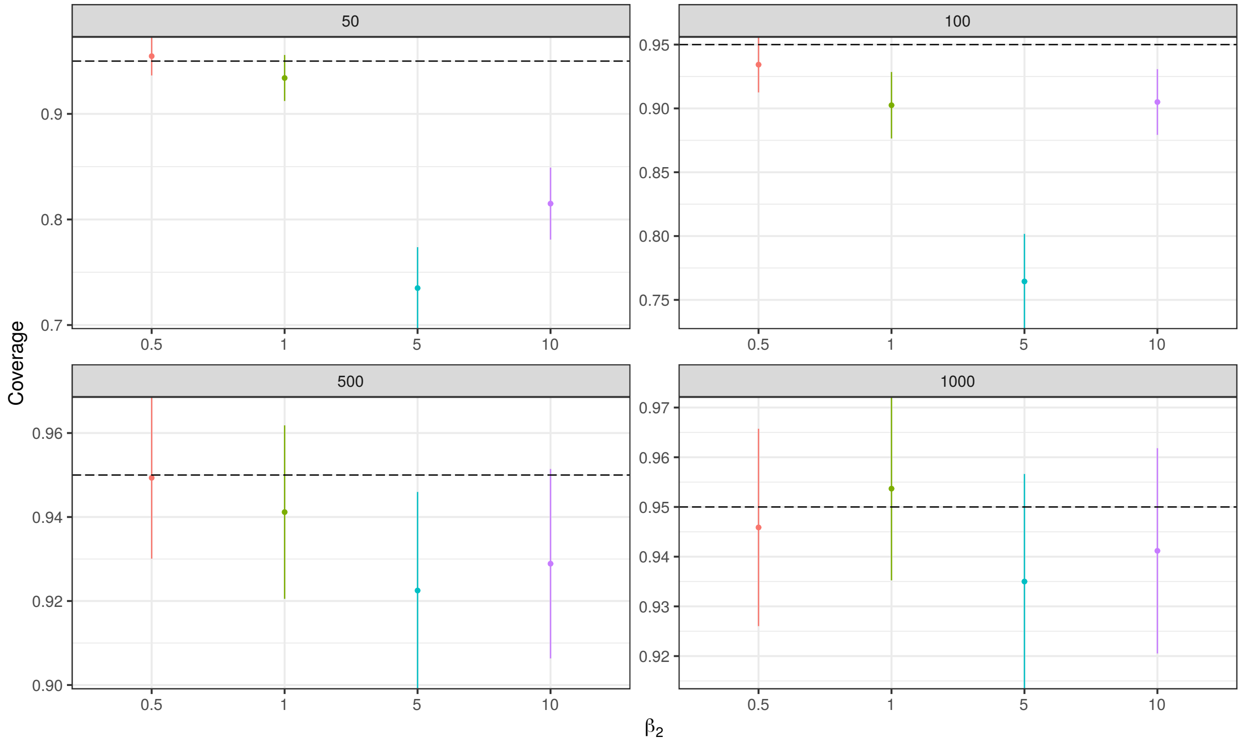

Figure B-5: **Coverage of 95% credibility intervals for β_2.** The horizontal dashed line marks the nominal coverage (0.95) and interval lines are 95% confidence intervals for the allowable Monte Carlo error.

For our sampling-aware scenarios, we apply our sampling time posterior predictive check as well. Our chi-squared sampling discrepancy D_{χ^2} generates a posterior predictive p-value of 0.72, correctly suggesting a good fit. The unrelated sampling scenario also produces a sampling posterior predictive p-value. We see a relatively low posterior predictive p-value of 0.15, reacting to differences between the true and inferred sampling intensity trajectories.

B.3 Sequence Data Inference Simulation Study

B.4 Validation of Estimation Procedure

Now, we expand the scope of our simulation study to be based on simulated sequence alignment data instead of a known genealogy. In this section, all of our examples will be based on an effective population size trajectory of $N_{e,1,10,1,0.5}(t)$, mimicking the trajectory of a seasonal disease as measured in units of years. Similar to the previous section, we generate sampling times and genealogies according to different sampling scenarios and the coalescent, respectively. Given a genealogy, we simulate sequence data using the software `SeqGen` [Rambaut and Grassly, 1997] using the Jukes-Cantor 1969 [Jukes et al., 1969] substitution model to generate 1500 sites. We use two substitution rates, high and medium, producing 0.9 and 0.09

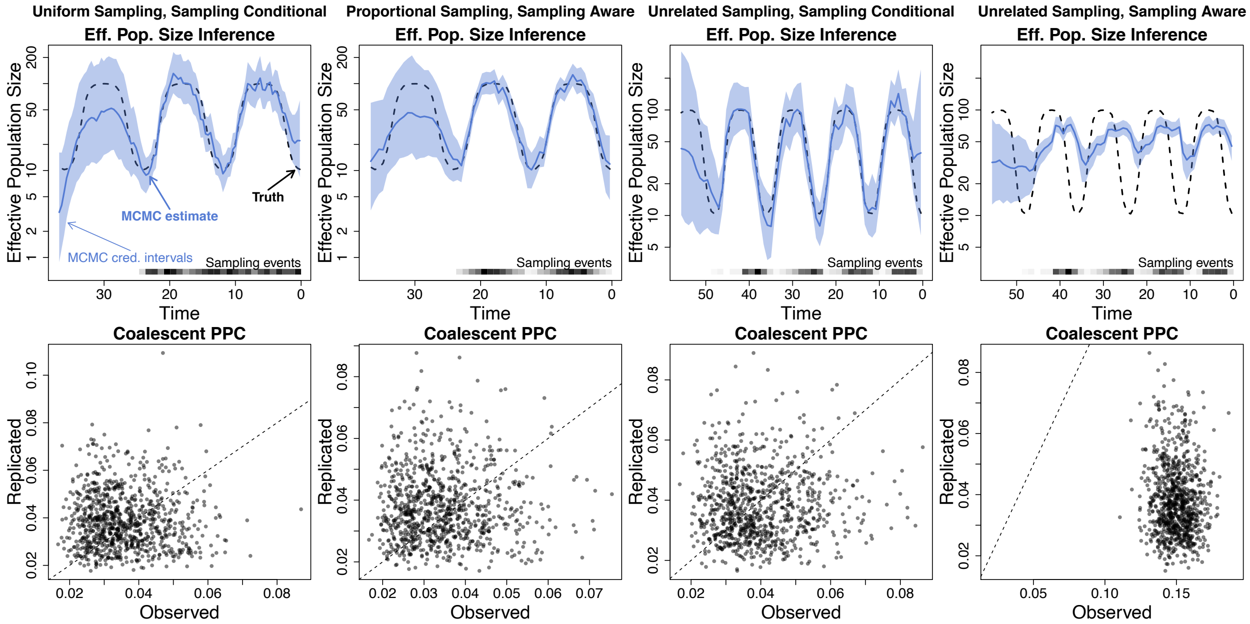

Figure B-6: **Effective population size inference and coalescent posterior predictive check for fixed-tree simulations.** The dashed black line represents the true effective population trajectory. The solid blue line represents the posterior median effective population trajectory inferred by fixed-tree MCMC and the light blue region represents the corresponding pointwise 95% credible intervals for the effective population trajectory.

Scenario	Sampling Model	Post. Pred. p-val	
		Coalescent	Sampling
Uniform	Conditional	0.58	—
Proportional	Aware: $\gamma(t)$	0.59	0.72
Unrelated	Conditional	0.46	—
Unrelated	Aware: $\gamma(t)$	0.00	0.15

Table B-1: **Posterior predictive p-values for simulated fixed-tree data.**

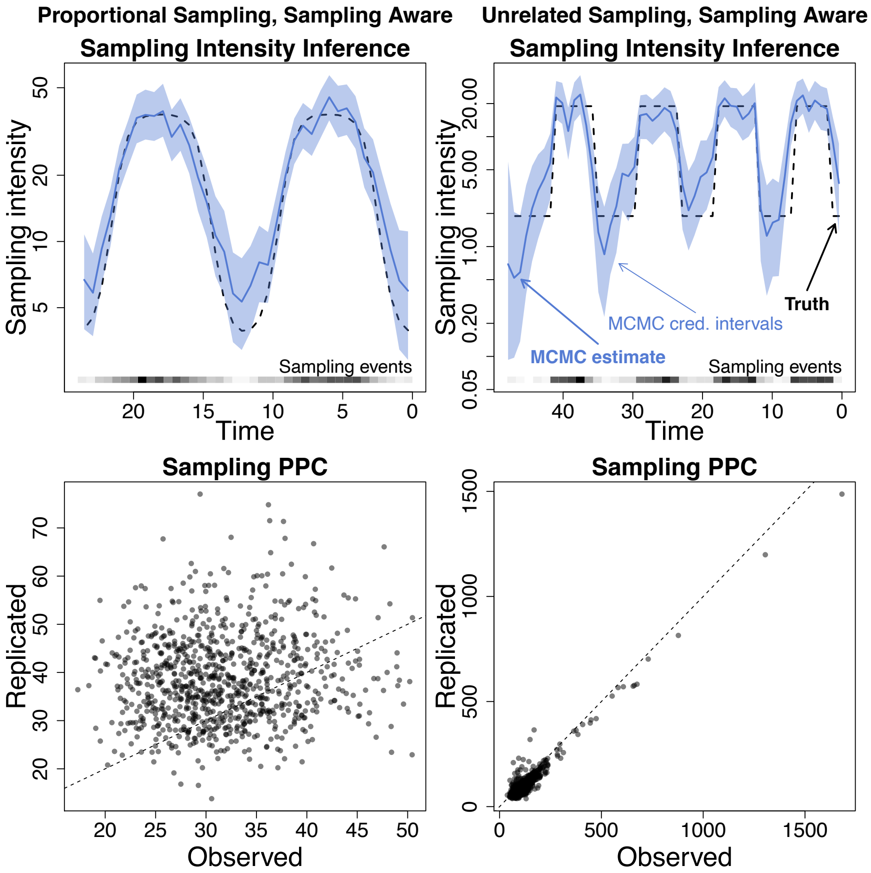

Figure B-7: **Sampling intensity inference and sampling time posterior predictive check for fixed-tree simulations.** The dashed black line represents the true sampling intensity. The solid blue line represents the posterior median sampling intensity inferred by fixed-tree MCMC, and the light blue region represents the corresponding pointwise 95% credible intervals for the sampling intensity.

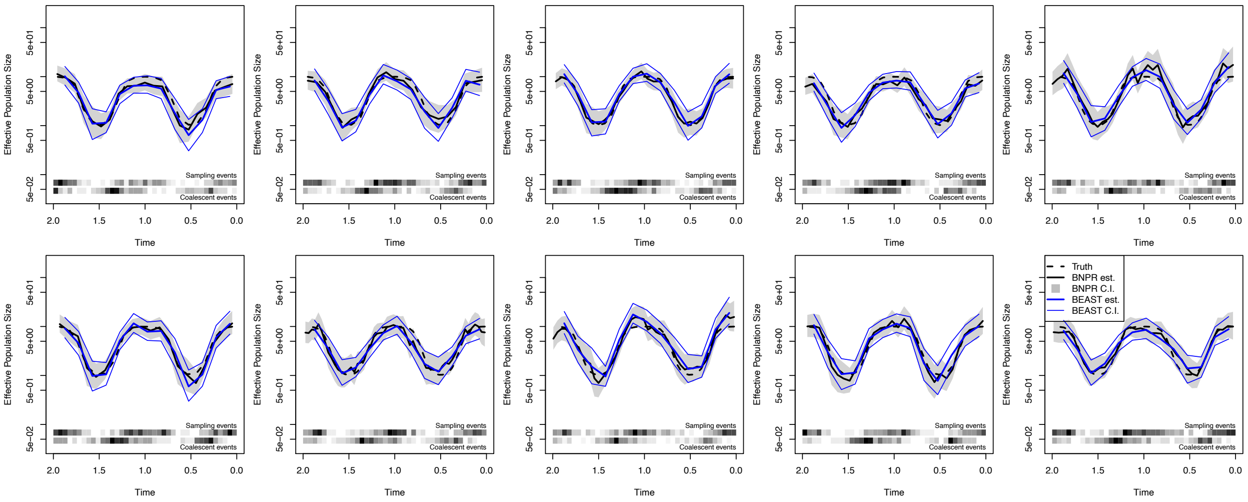

Figure B-8: **Effective population size inference for sequence data simulations: high substitution rate and no covariates.**

Scenario	Sampling Model	Post. Pred. p-val Coalescent	Post. Pred. p-val Sampling
Uniform	Conditional	0.51	—
Proportional	Conditional	0.50	—
Increasing	Aware: $\gamma(t)$	0.47	0.56
Unrelated	Aware: $\gamma(t)$	0.17	0.46

Table B-2: **Posterior predictive p-values for simulated sequence data.**

substitutions per site. We distribute 200 sampling times using preferential sampling model with no covariates and with one time covariate. We repeat each combination of high/medium and no covariates/one time covariate settings 10 times. We infer the underlying genealogy and effective population size trajectory using the software `BEAST` [Suchard et al., 2018] with an elliptical slice sampling transition kernel (ESS) [Murray et al., 2010] as implemented in Section 2, with a sampling-conditional posterior. Figures **??** show effective population estimation results of these simulations.

B.5 Testing Posterior Predictive Checks

Next, we use the same simulation set up to test our posterior predictive checking. Finally, we generate replicate genealogies as in the previous section, and we calculate our coalescent discrepancy D_c for the observed BEAST results as well as the replicates. In Figure B-12 (first column), we see that the effective population estimate is close to the true trajectory, and when we compare the observed and replicate discrepancies, we calculate a posterior predictive p-value of 0.51, corroborating the model's adequacy. Next, we distribute 170 sampling times between $t = 0$ and $t = 2$ (years) with sampling log-intensity $\log[\lambda_c(t)] = 2.90 + N_{e,1,10,1,0.5}(t)$.

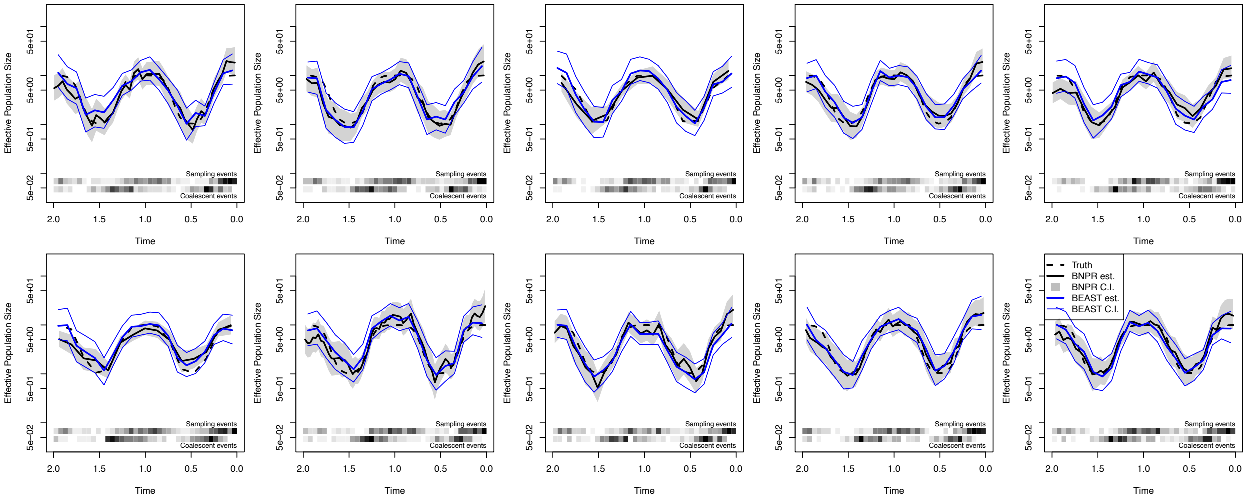

Figure B-9: **Effective population size inference for sequence data simulations: high substitution rate and one time covariate.**

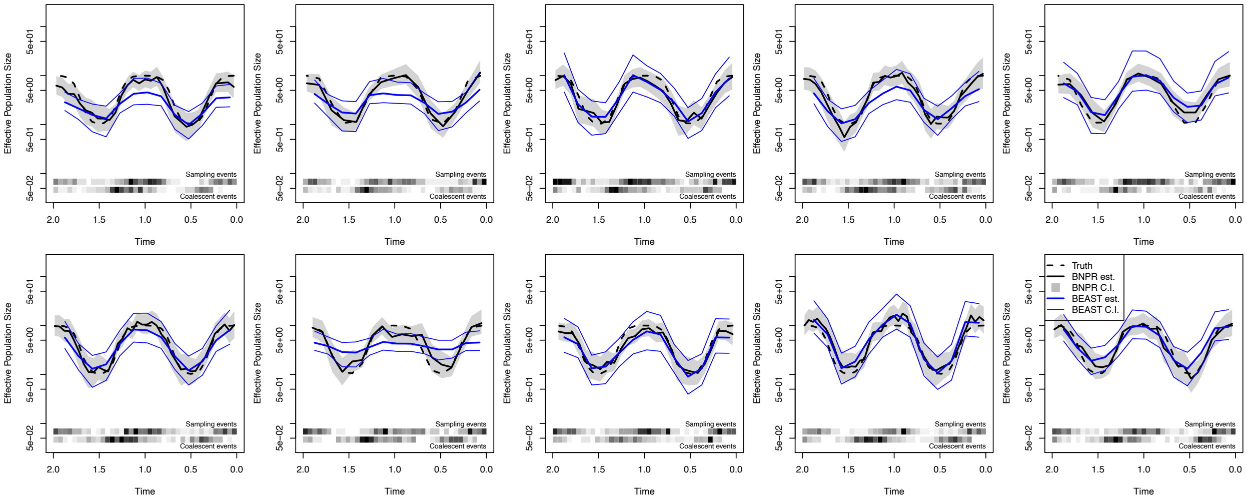

Figure B-10: **Effective population size inference for sequence data simulations: medium substitution rate and no covariates.**

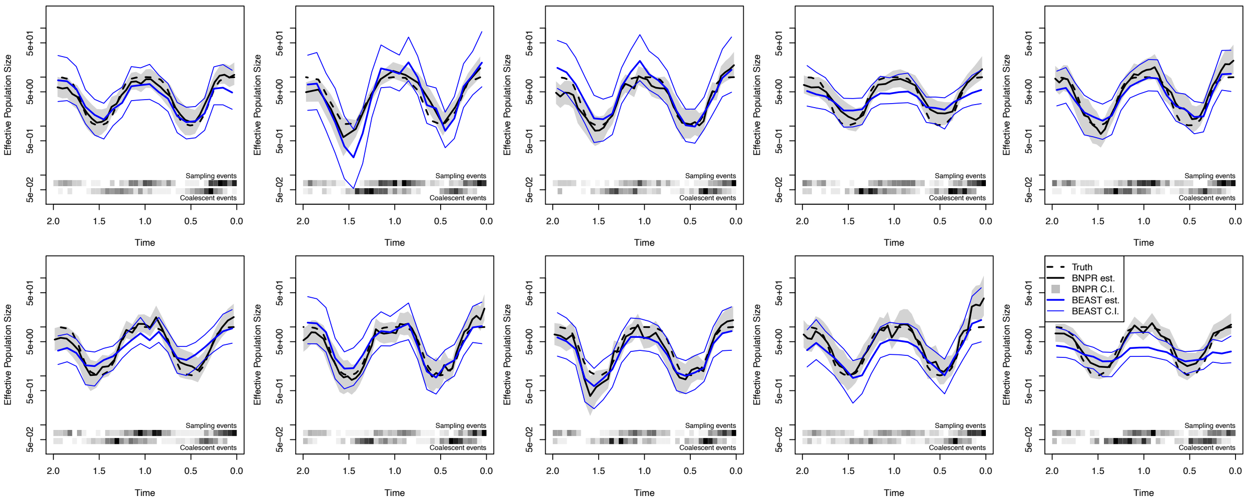

Figure B-11: **Effective population size inference for sequence data simulations: medium substitution rate and one time covariate.**

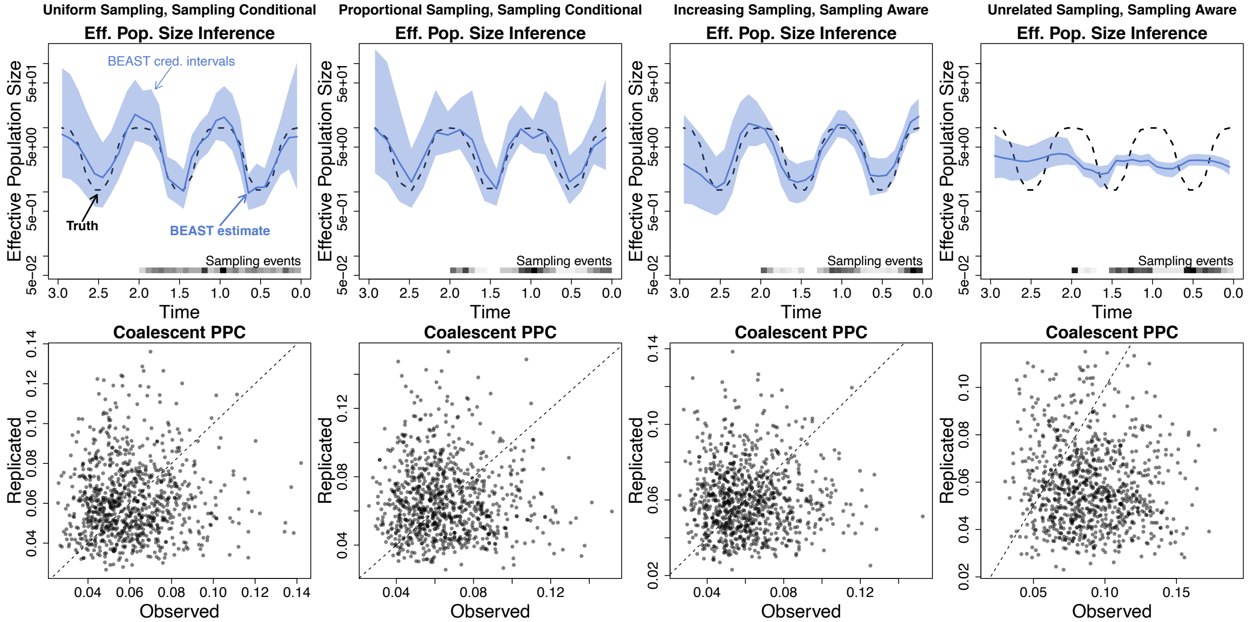

Figure B-12: **Effective population size inference and coalescent posterior predictive check for sequence data simulations.** The dashed black line represents the true effective population trajectory. The solid blue line represents the posterior median effective population trajectory inferred by BEAST, and the light blue region represents the corresponding pointwise 95% credible intervals for the effective population trajectory.

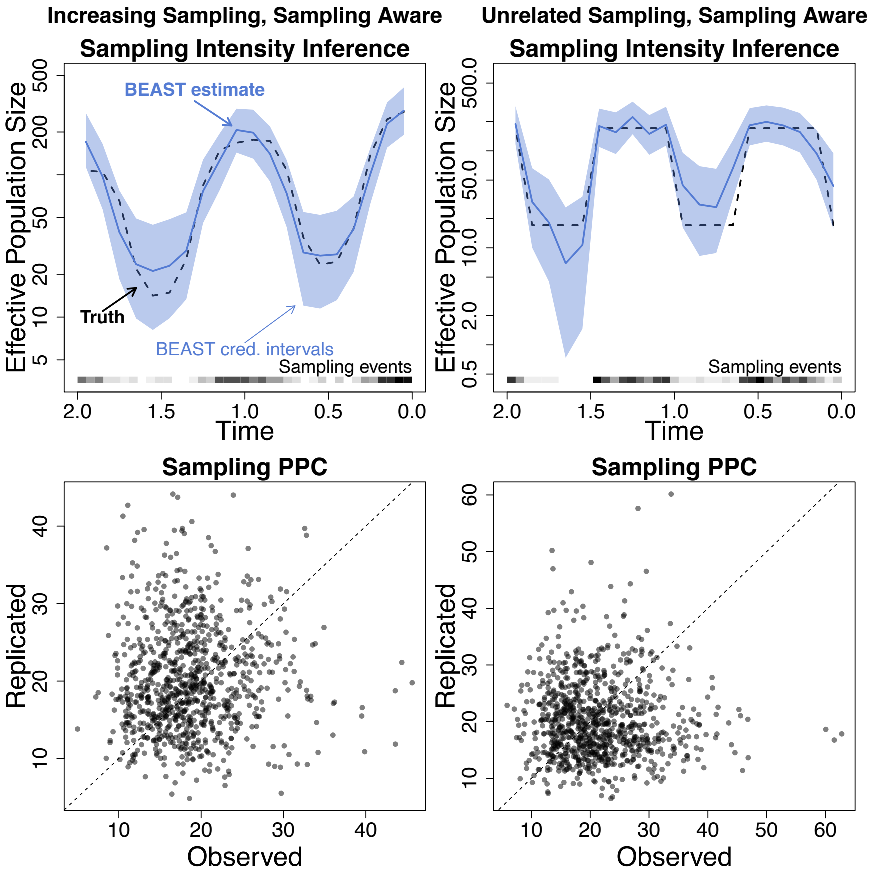

Figure B-13: **Sampling intensity inference and sampling time posterior predictive check for sequence data simulations.** The dashed black line represents the true sampling intensity. The solid blue line represents the posterior median sampling intensity inferred by BEAST, and the light blue region represents the corresponding pointwise 95% credible intervals for the sampling intensity.

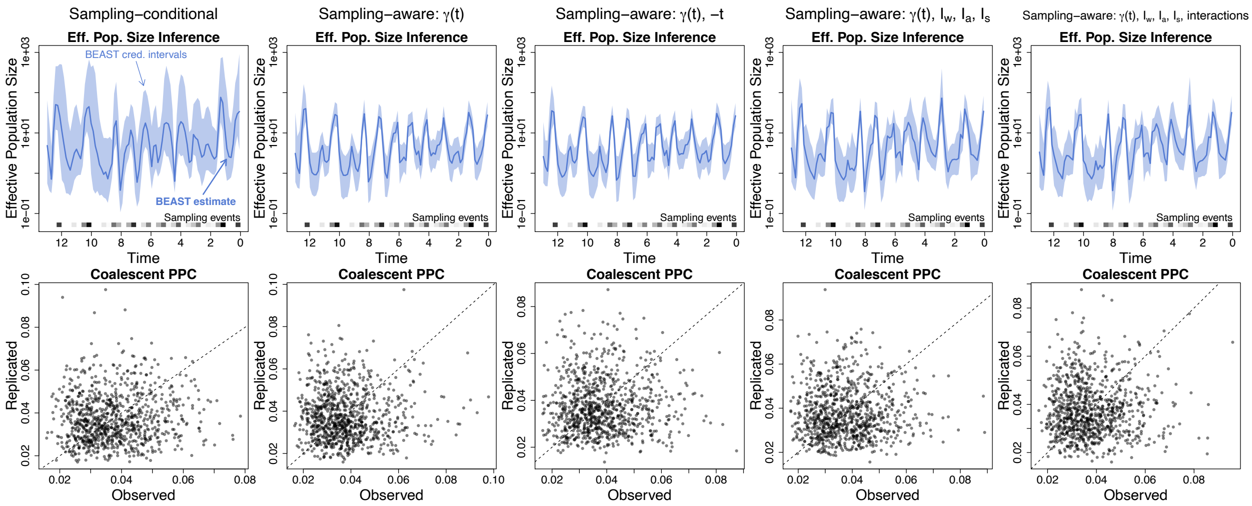

Figure B-14: **Effective population size inference and coalescent posterior predictive check for seasonal influenza data.** The solid blue line represents the posterior median effective population trajectory inferred by BEAST, and the light blue region represents the corresponding pointwise 95% credible intervals for the effective population trajectory.

We infer the underlying genealogy and effective population size trajectory using the sampling-conditional model and calculate discrepancies as above. Note this is a model misspecification applying a sampling-conditional model to a preferential sampling sampling scenario in the style of [Karcher et al., 2016]. Unfortunately, the posterior predictive p-value (0.50) does not detect this mismatch, as the bias effective population size estimate is hard to visually detect in Figure B-12.

In our third scenario, we distribute 199 sampling times between $t = 0$ and $t = 2$ (years) with increasing sampling log-intensity $\log[\lambda_c(t)] = 3.35 - 0.5t + N_{e,1,10,1,0.5}(t)$. We infer as above, but targeting the sampling-aware posterior with sampling log-intensity $\log[\lambda_s(t)] = \beta_0 + \beta_1 \cdot \boldsymbol{\gamma}(t)$. This is again a misspecification, as the model cannot recover the $-0.5t$ term. However, the posterior predictive check does not clearly detect the mismatch, with a posterior predictive p-value of 0.47. Our sampling posterior predictive check does not detect the misspecification either, with a posterior predictive p-value of 0.56. In our final scenario, we distribute 222 sampling times between $t = 0$ and $t = 2$ (years) with a sampling log-intensity $\log[\lambda_c(t)] = 2.84 + P'(t)$ ($P'(t)$ illustrated in Figure B-13, second column) unrelated to the effective population size. We target the sampling-aware posterior, with sampling log-intensity $\log[\lambda_s(t)] = \beta_0 + \beta_1 \cdot \boldsymbol{\gamma}(t)$. The model reconstructs the effective population size trajectory poorly, and this is successfully reflected in the posterior predictive p-value of 0.17. However, our sampling posterior predictive check does not detect the misspecification, with a posterior predictive p-value of 0.46.

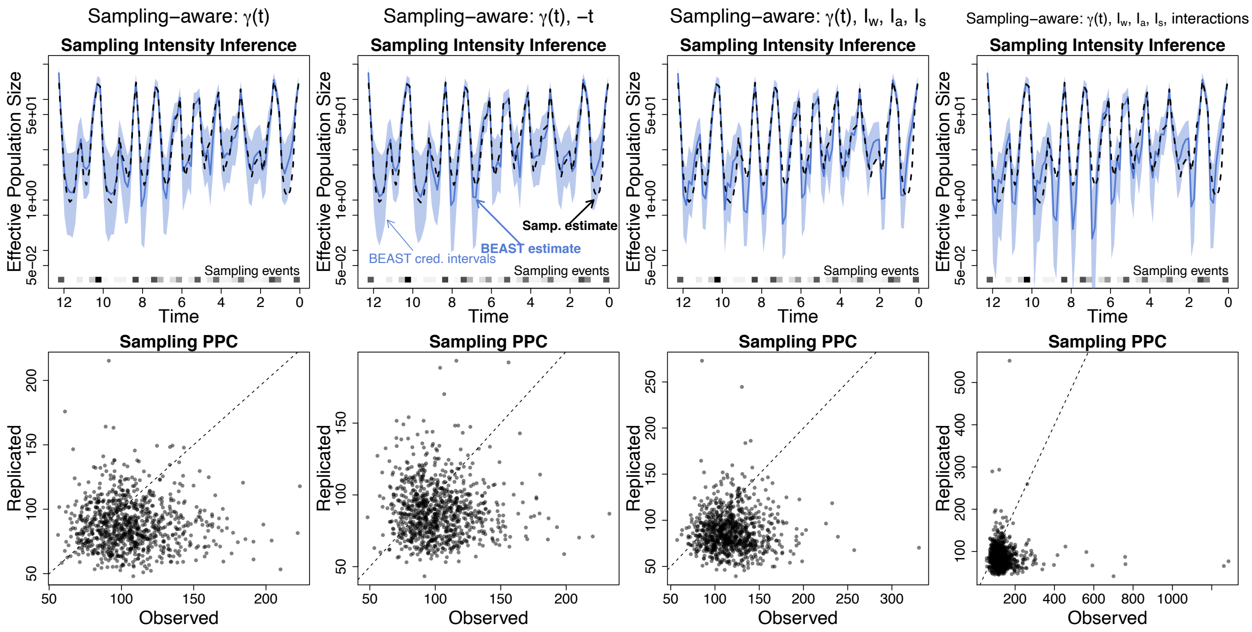

Figure B-15: **Sampling intensity inference and sampling time posterior predictive check for seasonal influenza data.** The dashed black line represents the true sampling intensity. The solid blue line represents the posterior median sampling intensity inferred by BEAST, and the light blue region represents the corresponding pointwise 95% credible intervals for the sampling intensity.

Sampling Model	Post. Pred. p-val	
	Coalescent	Sampling
Conditional	0.47	—
Aware: $\boldsymbol{\gamma}(t)$	0.48	0.29
Aware: $\boldsymbol{\gamma}(t), -t$	0.47	0.32
Aware: $\boldsymbol{\gamma}(t), I_w, I_a, I_s$	0.49	0.16
Aware: $\boldsymbol{\gamma}(t), I_w, I_a, I_s, \{I_w, I_a, I_s\} \cdot \boldsymbol{\gamma}(t)$	0.49	0.16

Table B-3: **Posterior predictive p-values for seasonal influenza data.**

B.5.1 Seasonal Influenza

We apply our posterior predictive check methods to the North American subset of global H3N2 influenza [Zinder et al., 2014]. The data contains 520 sequences aligned to form a multiple sequence alignment with 1698 sites of the hemagglutinin gene. We use the same sequence data `BEAST` framework as the previous section, choosing four different specific sampling time models. We use a sampling-conditional model with no sampling time model, a simple log-linear sampling time model, and sampling models with different sets of covariates, including $I_w(t) = I_{(t \bmod 1) \in [0, 0.25)}$ as an indicator function for winter, $I_a(t) = I_{(t \bmod 1) \in [0.25, 0.5)}$ as an indicator function for autumn, and $I_s(t) = I_{(t \bmod 1) \in [0.5, 075)}$ as an indicator function for summer.

Figure B-14 shows the inferred effective population size trajectories and coalescent posterior predictive checks for the models. All estimated trajectories follow a similar seasonal trajectory, and the discrepancy comparison suggests that the estimated trajectory produces reasonable results with large posterior predictive p-values (Table B-3). Figure B-15 shows the inferred sampling intensities compared against a nonparametric sampling time-only estimate of the sampling intensity, as well as sampling posterior predictive checks for the four models. The sampling posterior predictive check produces moderate-to-low posterior predictive p-values, suggesting some model inadequacy manifesting in the sampling intensity estimates.

B.5.2 Ebola Outbreak

Next, we analyze a subset of sequence data from the recent African Ebola outbreak [Dudas et al., 2017]. We use the same sequence data `BEAST` framework as the previous section, choosing four different specific sampling time models. We use a sampling-conditional model with no sampling time model, a simple log-linear sampling time model, and several additional sampling-aware models with different sets of covariates.

Figure B-16 shows the inferred effective population size trajectories and coalescent posterior predictive checks for the four models. All estimated trajectories follow a similar effective population size trajectory that visually resembles a typical time trajectory of prevalence or incidence that peaks in Autumn of 2014. The discrepancy comparison suggests that the estimated trajectory produces reasonable results with large posterior predictive p-values (Table

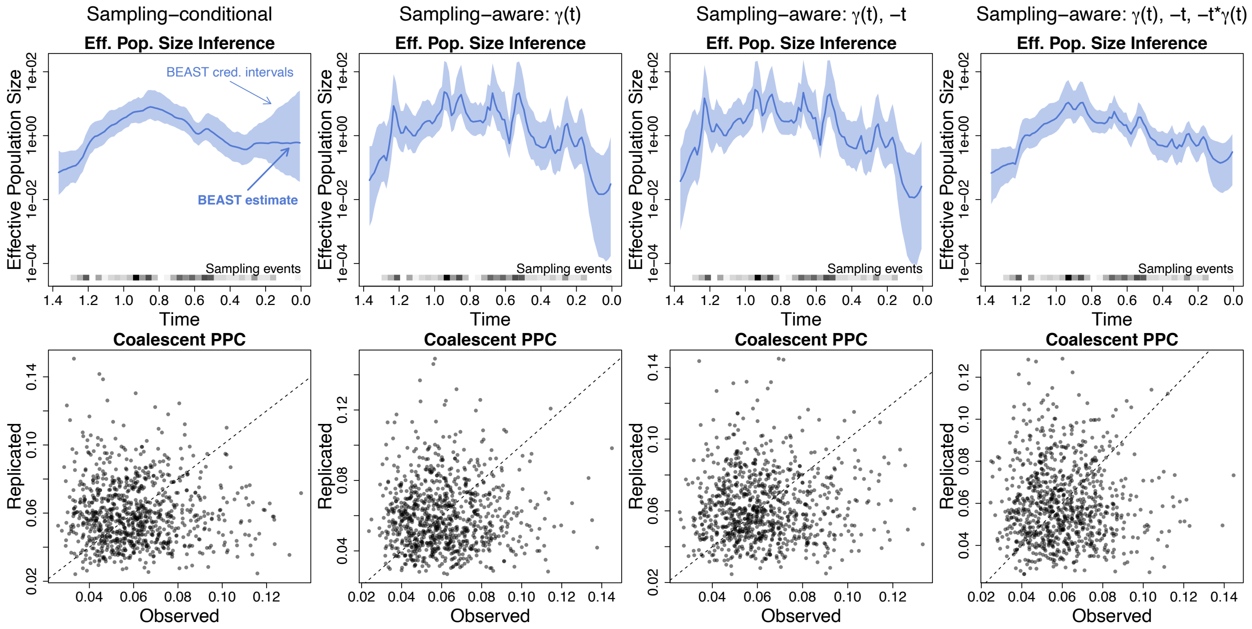

Figure B-16: **Effective population size inference and coalescent posterior predictive check for Sierra Leone Ebola data (part 1).** The solid blue line represents the posterior median effective population trajectory inferred by BEAST, and the light blue region represents the corresponding pointwise 95% credible intervals for the effective population trajectory.

B-4). Figure B-18 shows the inferred sampling intensities compared against a nonparametric sampling time-only estimate of the sampling intensity, as well as sampling posterior predictive checks for the four models. The sampling posterior predictive check produces small posterior predictive p-values (Table B-4), suggesting notable model inadequacy manifesting in the sampling intensity estimates.

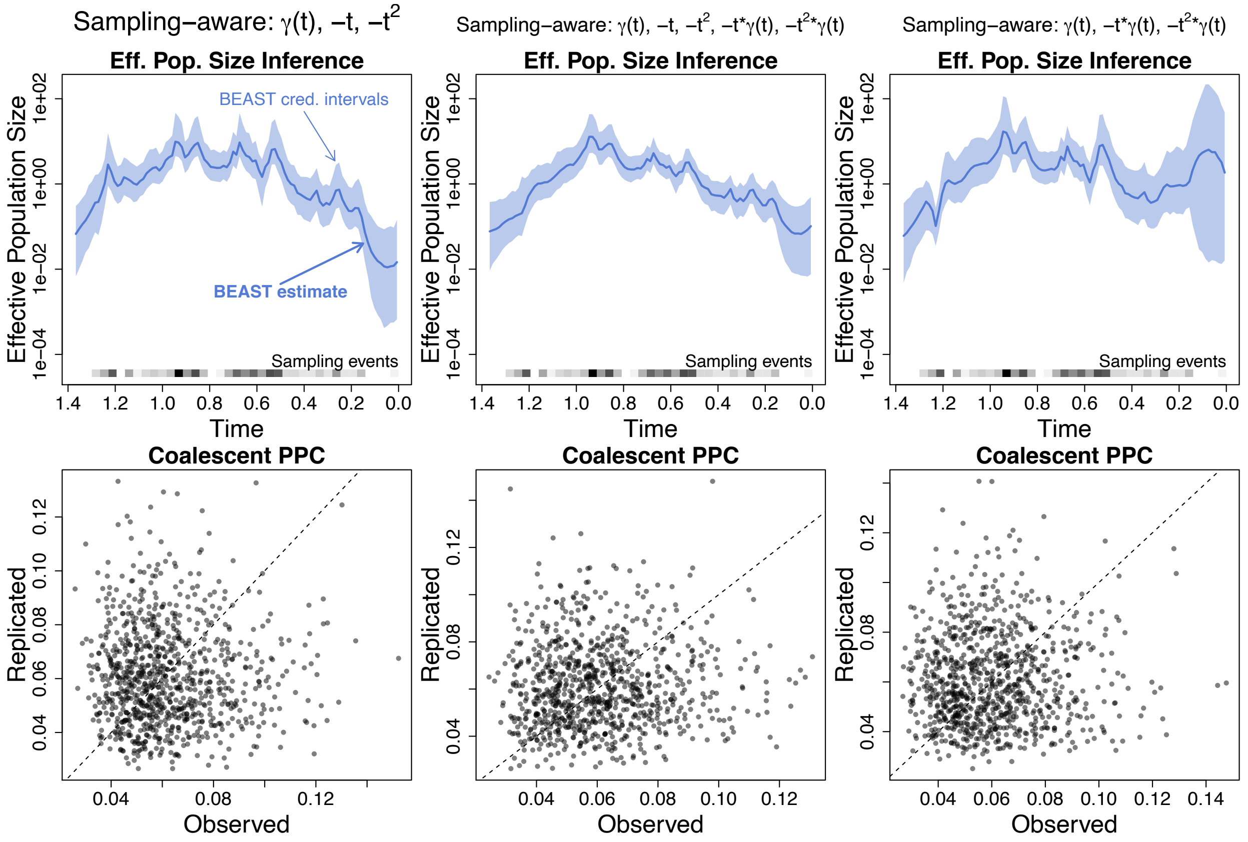

Figure B-17: **Effective population size inference and coalescent posterior predictive check for Sierra Leone Ebola data (part 2).** The solid blue line represents the posterior median effective population trajectory inferred by BEAST, and the light blue region represents the corresponding pointwise 95% credible intervals for the effective population trajectory.

**Sampling Model**	**Post. Pred. p-val**	
	Coalescent	Sampling
Conditional	0.48	—
Aware: $\boldsymbol{\gamma}(t)$	0.47	0.15
Aware: $\boldsymbol{\gamma}(t), -t$	0.50	0.18
Aware: $\boldsymbol{\gamma}(t), -t, -t \cdot \boldsymbol{\gamma}(t)$	0.50	0.06
Aware: $\boldsymbol{\gamma}(t), -t, -t^2$	0.48	0.31
Aware: $\boldsymbol{\gamma}(t), -t, -t^2, \{-t, -t^2\} \cdot \boldsymbol{\gamma}(t)$	0.51	0.17
Aware: $\boldsymbol{\gamma}(t), \{-t, -t^2\} \cdot \boldsymbol{\gamma}(t)$	0.51	0.22

Table B-4: **Posterior predictive p-values for Sierra Leone Ebola data.**

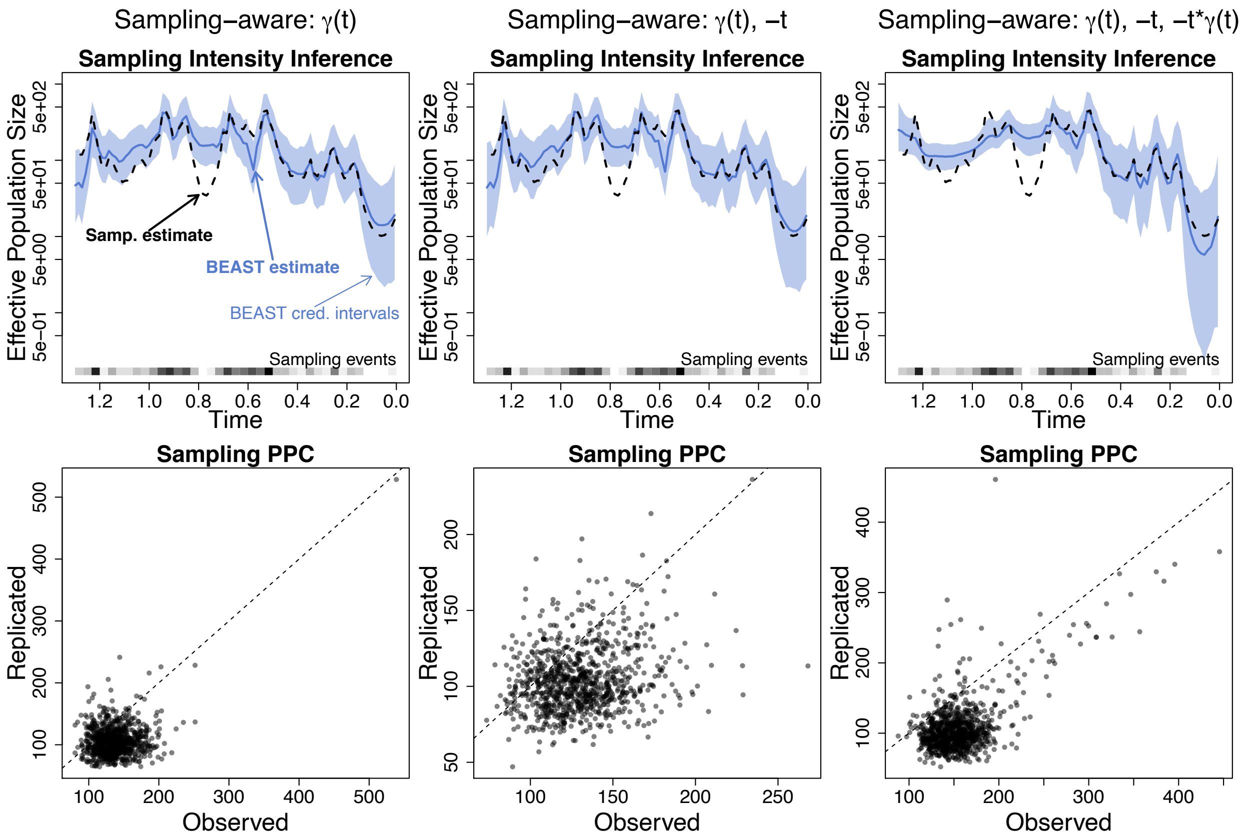

Figure B-18: **Sampling intensity inference and sampling time posterior predictive check for Sierra Leone Ebola data (part 1).** The dashed black line represents the true sampling intensity. The solid blue line represents the posterior median sampling intensity inferred by BEAST, and the light blue region represents the corresponding pointwise 95% credible intervals for the sampling intensity.

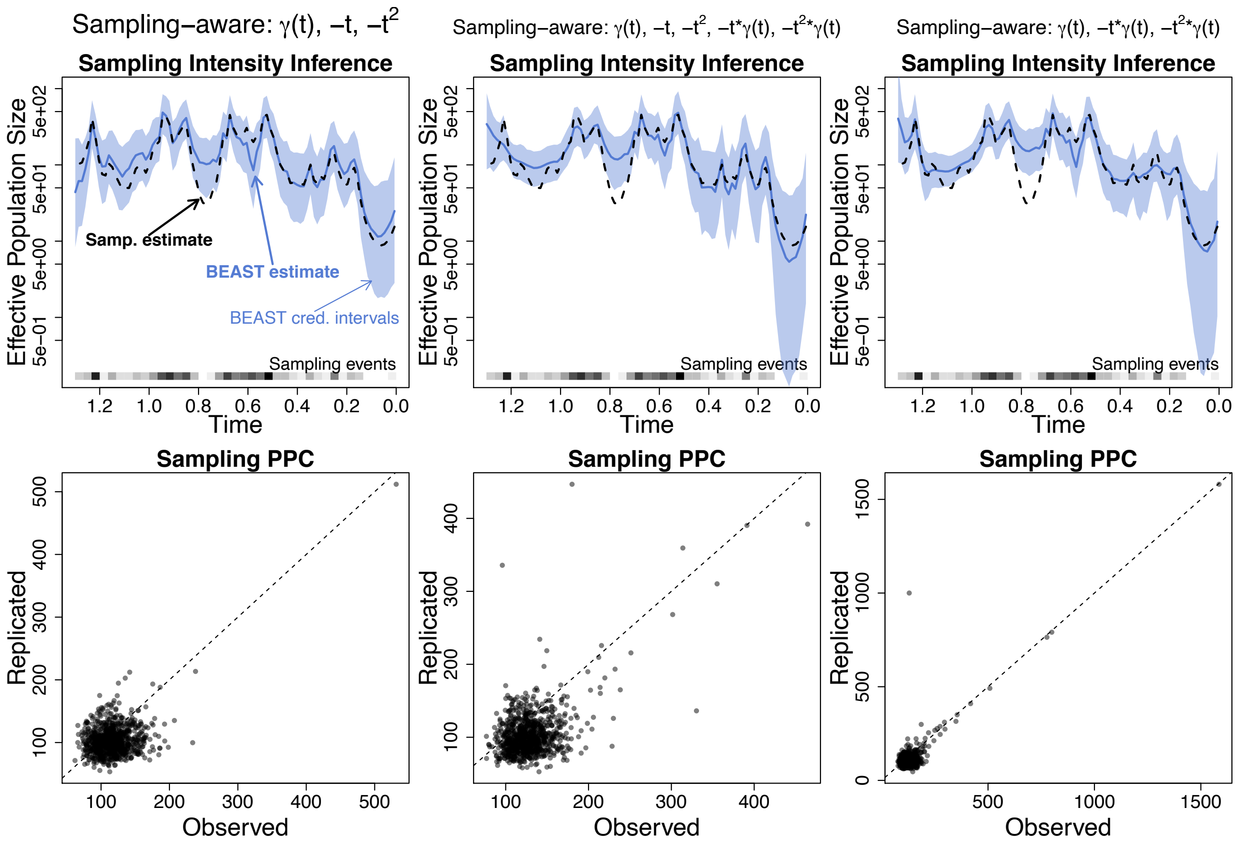

Figure B-19: **Sampling intensity inference and sampling time posterior predictive check for Sierra Leone Ebola data (part 2).** The dashed black line represents the true sampling intensity. The solid blue line represents the posterior median sampling intensity inferred by BEAST, and the light blue region represents the corresponding pointwise 95% credible intervals for the sampling intensity.

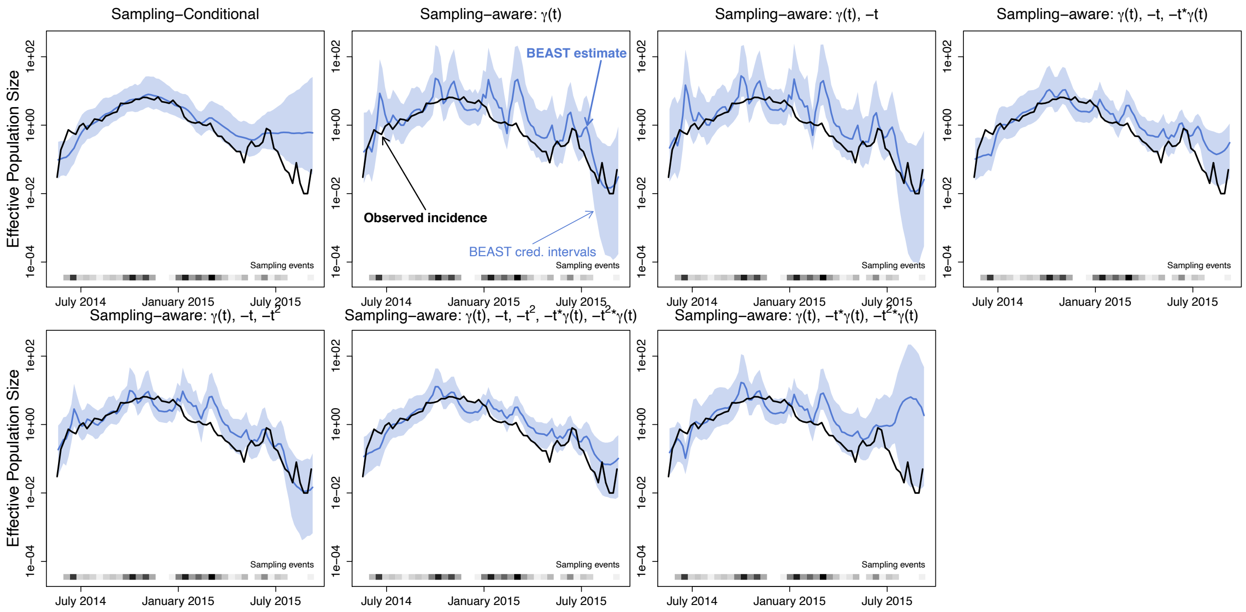

Figure C-1: **Comparison of effective population size reconstructions with incidence data in Sierra Leone.** The solid blue line represents the posterior median effective population trajectory inferred by BEAST, and the light blue region represents the corresponding pointwise 95% credible intervals for the effective population trajectory. The solid black line shows the observed incidence scaled by a constant so it is on the same scale as the effective population size.

C Appendix: Ebola incidence data

As we explain in Section 4.3, we compare estimated effective population size trajectories with observed incidence data. We multiplied incidence count by 0.01 — a number determined by trial-and-error, to bring incidence and effective population size to the same scale — and plot effective population size posterior summaries and incidence counts for Sierra Leone and Liberia in Figures C-1 and C-2.

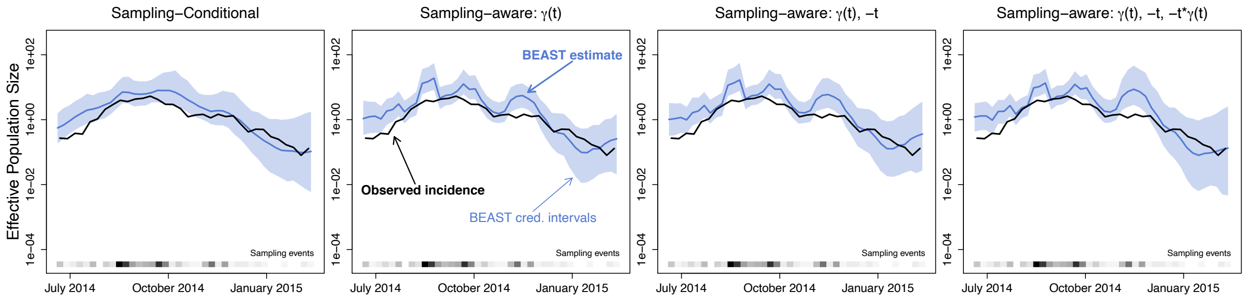

Figure C-2: **Comparison of effective population size reconstructions with incidence data in Liberia.** See Figure C-1 caption for the legend explanation.
